# Molecular Simulations and Mechanistic Analysis of the Effect of CO_2_ Sorption on Thermodynamics, Structure, and Local Dynamics of Molten Atactic Polystyrene

**DOI:** 10.1021/acs.macromol.0c00323

**Published:** 2020-05-11

**Authors:** Eleonora Ricci, Niki Vergadou, Georgios G. Vogiatzis, Maria Grazia De Angelis, Doros N. Theodorou

**Affiliations:** † Department of Civil, Chemical, Environmental and Materials Engineering, University of Bologna, Bologna, Italy; ‡ Institute of Nanoscience and Nanotechnology, National Center for Scientific Research “Demokritos”, Athens, Greece; § Department of Mechanical Engineering, Eindhoven University of Technology, Eindhoven, The Netherlands; ∥ School of Chemical Engineering, National Technical University of Athens, Athens, Greece

## Abstract

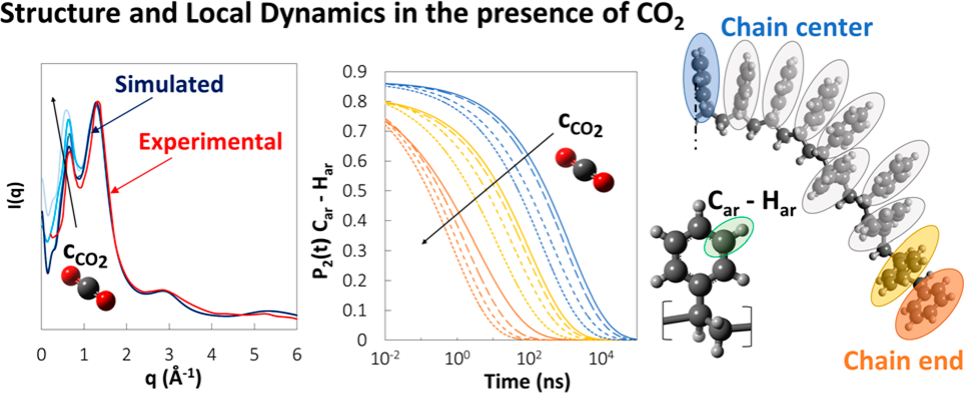

A simulation strategy encompassing different scales was applied to the systematic study of the effects of CO_2_ uptake on the properties of atactic polystyrene (aPS) melts. The analysis accounted for the influence of temperature between 450 and 550 K, polymer molecular weights (*M*_w_) between 2100 and 31000 g/mol, and CO_2_ pressures up to 20 MPa on the volumetric, swelling, structural, and dynamic properties of the polymer as well as on the CO_2_ solubility and diffusivity by performing molecular dynamics (MD) simulations of the system in a fully atomistic representation. A hierarchical scheme was used for the generation of the higher *M*_w_ polymer systems, which consisted of equilibration at a coarse-grained level of representation through efficient connectivity-altering Monte Carlo simulations, and reverse-mapping back to the atomistic representation, obtaining the configurations used for subsequent MD simulations. Sorption isotherms and associated swelling effects were determined by using an iterative procedure that incorporated a series of MD simulations in the *NPT* ensemble and the Widom test particle insertion method, while CO_2_ diffusion coefficients were extracted from long MD runs in the *NVE* ensemble. Solubility and diffusivity compared favorably with experimental results and with predictions of the Sanchez–Lacombe equation of state, which was reparametrized to capture the *M*_w_ dependence of polymer properties with greater accuracy. Structural features of the polymer matrix were correctly reproduced by the simulations, and the effects of gas concentration and *M*_w_ on structure and local dynamics were thoroughly investigated. In the presence of CO_2_, a significant acceleration of the segmental dynamics of the polymer occurred, more pronouncedly at low *M*_w_. The speed-up effect caused by the swelling agent was not limited to the chain ends but affected the whole chain in a similar fashion.

## Introduction

Supercritical CO_2_ has found many applications in the fabrication and processing of polymers to selectively control and manipulate their physical properties by changing temperature and pressure, taking advantage of the fact that its critical point (304.2 K and 7.4 MPa^1^) allows operating at mild conditions. Among several applications, supercritical CO_2_ is used as a reaction solvent and as an extraction medium, especially in the food and pharmaceutical industries, because of its nontoxic nature. It is also applied in the impregnation of polymers to introduce dyes, antibacterial or antioxidant substances, or other types of additives into the matrix. Additionally, it is employed in fractionation, foaming, blending, particle formation, and injection molding,^2^ usually exploiting the swelling and plasticization of the polymer resulting from the dissolution of large amounts of CO_2_ into the matrix. Moreover, the analysis of CO_2_/polymer systems at high temperatures is of practical interest for membrane separation processes for precombustion CO_2_ capture^3^ and in the development of flexible materials for controlled atmosphere packaging capable of withstanding sterilization conditions to replace tin cans and glass containers.^4^ Indeed, the addition of compressed CO_2_ to a condensed phase is responsible for substantial changes in the physical properties of interest during processing, such as viscosity, permeability, interfacial tension, and glass transition temperature. A detailed understanding of the effects of CO_2_ on polymer properties is therefore of great interest in a wide range of industrial sectors.

Atactic polystyrene (aPS) is one of the most common plastic materials, ubiquitously used in manufacturing and packaging, often processed by using supercritical CO_2_ as a blowing agent, up to high temperatures.^5,6^ For this reason, a rich characterization of its properties has been performed over the years, including its gas transport properties and the effect of CO_2_ on its thermodynamic, structural, and mechanical properties, both in the glassy and in the melt state. In particular, sorption and diffusion of CO_2_ in glassy aPS were measured by several authors, assessing the effects of molecular weight^7^ and chain orientation^8^ on sorption and swelling and identifying the onset of devitrification induced by the gas as a function of temperature.^9^ In addition, CO_2_ sorption and swelling have been studied in polymer blends of aPS with poly(2,6-dimethyl-1,4-phenylene oxide) (PPO),^10^ polycarbonate,^11^ block copolymers containing styrene,^12^ and poly(methyl methacrylate) (PMMA).^13^ More recently, Pantoula et al.^14,15^ performed an extensive characterization of the aPS-CO_2_ system in terms of gas sorption and swelling in the range 35–132 °C and up to 45 MPa. They also applied the nonrandom hydrogen-bonding (NRHB) model^16,17^ to represent the behavior of the system in this wide range of conditions.

Fewer studies were devoted to the characterization of the aPS-CO_2_ system in the melt state. Newitt and Weale^18^ reported a few values of CO_2_ solubility and diffusivity in aPS melt at high temperature and pressure. Hilic et al.^19^ performed simultaneous measurement of sorption and associated swelling effects for nitrogen and carbon dioxide in polystyrene from 65 to 130 °C and up to 45 MPa. Areerat et al.^20^ extended the range of available data by studying CO_2_ sorption in polystyrene with a gravimetric technique in the range 150–200 °C up to 12 MPa. Moreover, they determined CO_2_ diffusion coefficients from the analysis of sorption kinetics and modeled the system by using the Sanchez–Lacombe EoS. Sato et al.^21−23^ worked extensively on the aPS–CO_2_ systems, obtaining sorption isotherms as well as diffusion coefficients in the melt state up to 200 °C and 20 MPa, using both manometric and gravimetric techniques. More recently, Perez-Blanco et al.^24^ determined CO_2_ solubility and diffusivity in aPS, in both the glassy and the melt state, from 30 to 200 °C and up to 8.5 MPa, finding good agreement with previous works.

The use of molecular simulations for the prediction of material properties has experienced an impressive outburst in recent years due to the increase in the computational power and the development and optimization of new efficient algorithms and methods, capable of addressing larger systems, wider length and time scale phenomena, and more complex chemical structures.^25−28^ These methods constitute unique means to gain insight into the microscopic structure and dynamics of materials and to perform predictive analyses under conditions that cannot be accessed experimentally. Concerning molecular modeling, aPS is one of the benchmark systems for methodological development, and there is a large body of works devoted to reproducing the properties of the pure polymer,^29−34^ nanocomposite aPS–filler systems,^35−38^ polymer blends,^39^ and also gas-polymer systems by using molecular dynamics (MD), Monte Carlo (MC) simulations, or a combination of both. In particular, Cuthbert et al.^40^ used an all-atom representation to study system size effects on the calculated excess chemical potential of gases in amorphous glassy polystyrene at 300 K. Their results showed that small system sizes are, on average, unable to form cavities of sufficient size to accommodate gases of the size of CO_2_, resulting in insufficient sampling with Widom’s test particle insertion method.^41^ They found that for the case of CO_2_ in aPS it is required to have at least a single chain of 364 units (box side length 40 Å) to obtain a reasonable value of the excess chemical potential. Kucukpinar et al.^42^ applied the transition state approach^43^ instead, using a spherical representation of the penetrant and allowing only an elastic motion for the polymer matrix. They determined diffusion coefficients that in the case of CO_2_ in aPS at 300 K were 1 order of magnitude lower than the experimental values, whereas solubility values were in significantly better agreement, higher by a factor ranging between 1.5 and 2.8. Eslami et al.^44^ used a grand-canonical ensemble MD, namely the grand equilibrium MD method,^45^ to evaluate infinite dilution solubility coefficients up to 500 K and sorption isotherms up to high pressures at 298 and 373 K, in good agreement with experimental measurements. Mozaffari et al.^46^ combined infinite dilution coefficients and diffusivities in the zero pressure limit, evaluated through the mean-squared displacement of gas molecules during MD trajectories, to obtain estimates of the permeability coefficients of several light gases in aPS, including CO_2_, in the range 300–500 K. Spyriouni et al.^47^ calculated CO_2_ sorption isotherms up to high pressures both in the glassy and in the low-temperature melt state by means of an iterative procedure comprising *N*_1_*N*_2_*PT* MD simulations and the direct particle deletion (DPD) scheme for the calculation of gas fugacity inside the matrix coupled to EoS modeling with the PC-SAFT EoS to find the corresponding pressure of the system at a given concentration and fugacity. Their results were in very good agreement with experimental measurements in terms of both sorption and polymer swelling.

Despite the large number of studies focusing on the properties of systems formed by CO_2_ and polystyrene, the range of conditions inspected is relatively narrow. Indeed, most of the reports are focused on a single temperature, infinite dilution conditions, or monodisperse systems at a single molecular weight (*M*_w_). The present work aims at a comprehensive understanding of temperature (*T*), gas concentration (*c*_CO_2__) or, correspondingly, pressure (*P*), and polymer molecular weight effects on the system properties in the melt state. In the temperature range under study, system dynamics is fast enough to be accessible to MD simulations. Moreover, this work extends beyond the temperature range investigated with a similar scheme in ref (47), in which a maximum temperature of 405 K was reached.

Atomistic and coarse-grained methods were applied synergistically, in particular for the higher *M*_w_ studied: in this case a multiscale strategy was applied for the generation of equilibrated initial configurations,^35^ which involves the implementation of a coarse-grained model for aPS, the equilibration of the system at the coarse-grained level using connectivity altering MC,^48,49^ and reverse mapping to the atomistic level of description. Sorption isotherms have been evaluated up to high concentrations by using an iterative scheme similar to the one presented previously,^47^ and swelling effects have been studied. Gas diffusion coefficients have been extracted from the mean-squared displacement of gas molecules during long MD runs, and the effects of *T*, gas concentration, and *M*_w_ on the volumetric properties, local structural features, and local dynamics of the system have been extensively analyzed.

Equations of state (EoS) predictions using the Sanchez–Lacombe equation of state (SL EoS)^50^ were also used to compare with molecular simulation results at conditions not characterized experimentally. To represent sorption and swelling accurately at all molecular weights, a *M*_w_-dependent parametrization of the EoS was performed, based on experimental *pVT* data for the polymer. Subsequently, the CO_2_–aPS binary interaction parameter required for predicting binary properties was determined from the best fit of experimental sorption data.

## Methodology

### Generation of Initial Configurations

Monodisperse melts of atactic polystyrene of different molecular weights (*M*_w_) were studied, consisting of (a) 5 chains of 300 repeating units (∼31000 g/mol), (b) 30 chains of 50 repeating units (∼5200 g/mol), and (c) 75 chains of 20 repeating units (∼2100 g/mol). Table 1 summarizes the specifics of each pure polymer system and the CO_2_ concentration values used for the simulation of aPS/CO_2_ mixtures at the three different temperatures. To choose these concentration values, three values for the pressure were selected (2, 10, and 20 MPa), and the corresponding concentrations predicted with the Sanchez–Lacombe EoS at 450, 500, and 550 K were calculated and adopted throughout the simulations at all *M*_w_. The simulation box edge length was between 64 and 67 Å for all systems, and the number of atoms in each system was around 24000. Simulations were performed in full atomistic detail during all equilibration and production runs for the two lower *M*_w_ systems. The highest molecular weight initial configurations (300 repeating units) were generated according to the coarse-graining/equilibration/reverse-mapping strategy described in previous works.^32,35,47^ The coarse-grained representation adopted, specifically developed for the study of vinyl polymers,^29^ groups all atoms of a repeating unit into one superatom, mapping the polymer to a linear sequence of beads. Depending on the configuration of pairs of consecutive methylene backbone carbons, a sequence of meso and racemo dyads can be unequivocally defined and used to keep track of the stereochemistry of the chain. At first, a coarse-grained initial configuration is generated by growing chains within the primary simulation box by following the quasi-Metropolis bond-by-bond growth scheme of Theodorou and Suter,^51^ and then meso and racemo identities are assigned to every coarse-grained site by sampling a Bernoullian distribution. The system is then equilibrated at the coarse-grained level through a Monte Carlo simulation, making use of connectivity altering moves.^48,49^ Following equilibration, the system is back-mapped to the target all-atom representation for final equilibration at the atomistic level and production runs. This multiscale strategy has proven to be very effective in equilibrating polystyrene melts up to 4000 repeating units.^35^ The intermediate and lower *M*_w_ systems were generated by using the rotational isomeric state (RIS) model^52^ as modified by Theodorou and Suter^51^ and directly equilibrated at the atomistic level. All atomistic MD simulations were performed with the LAMMPS package.^53^

**Table 1:** Number of Atoms and *M*_w_ of the Systems Studied as Well as CO_2_ Concentration at Each *T*

system	*M*_w_ (g/mol)	number of atoms
75 chains × 20 repeating units	2099	24375
30 chains × 50 repeating units	5223	24150
5 chains × 300 repeating units	31261	24025

concentration (g_CO2_/g_pol_)	*T* = 450 K	*T* = 500 K	*T* = 550 K
low	7.00 × 10^–3^	5.70 × 10^–3^	5.00 × 10^–3^
intermediate	3.65 × 10^–2^	2.82 × 10^–2^	2.40 × 10^–2^
high	6.87 × 10^–2^	5.05 × 10^–2^	4.08 × 10^–2^

### Simulation Details

The potential energy form and the parameters for the all-atom (AA) representation of aPS were adopted from the work of Müller-Plathe,^54^ in conjunction with harmonic constants for bond stretching from the work of Ndoro et al.,^36^ while the EPM2 model was chosen for CO_2_.^55^ Lorentz–Berthelot mixing rules were applied for nonbonded interactions between unlike atoms of the polymer. A geometric mean combining rule was adopted for C_CO_2__–O_CO_2__ nonbonded interactions, for both ε and σ.^55^ For interactions between CO_2_ and polystyrene atoms, Lorentz–Berthelot mixing rules were applied. In the representation of CO_2_, bond angle deformations were described with *E*(ϕ) = *K*(1 + cos ϕ) instead of an harmonic potential of the form *E*(ϕ) = (*K*/2)(ϕ – ϕ_0_)^2^, as suggested by Müller-Plathe,^56^ to overcome the singularity present in the harmonic potential when the angle ϕ becomes 180°, and the equilibrium value ϕ_0_ is also 180°, which is the case for CO_2._ All parameters are reported in the Supporting Information (Tables S1–S3).

The system was simulated with periodic boundary conditions by using the rRESPA multi-timescale integrator,^57^ with two levels: at the innermost level a time step of 0.5 fs was employed to compute all bonded and short-range nonbonded interactions, while long-range electrostatic interactions were computed every 1 fs. For temperature and pressure control, the Nosé–Hoover thermostat and the barostat by Shinoda et al. were used^58,59^ with a damping parameter of 100 fs for temperature relaxation and 1000 fs for pressure relaxation. Nonbonded interactions were excluded between first and second bonded neighbors. A cutoff of 12 Å was used, and a pairwise neighbor list of 14 Å of radius was updated every 5 time steps. Tail corrections were applied to account for the long-range van der Waals interactions, and long-range electrostatics were computed with a particle–particle particle-mesh (pppm) method^60^ with a relative error in forces evaluation of 10^–6^.

The three different molecular weight systems were generated at 500 K for the pure polymer. At first, energy minimization was performed, by using the conjugate gradient method, to remove close contacts between atoms originated during the system generation or the back-mapping procedure. Afterward, to obtain initial configurations at different temperatures (450 and 550 K), the system was heated or cooled to the target temperature by applying a temperature ramp of 10 K/ns. The effect of the cooling rate on the final density of the systems was checked by applying a temperature ramp 5 times slower (2 K/ns) and 5 times faster (50 K/ns). The results obtained are reported in Figure S4: it was thereby verified that the cooling rate, in the range inspected, slightly affected the final density of the systems and not in a systematic way, and the individual density values at different cooling rates were estimated within the calculated uncertainty. The systems were then equilibrated at each temperature with 5 ns *NVT* runs followed by a 20 ns *NPT* run and a second 20 ns *NVT* run. Afterward, the systems were simulated for 50–100 ns in the *NVE* ensemble, depending on the relaxation times of the local dynamics: at lower temperature and high molecular weight longer times are required to observe decorrelation of the system from its initial state. During the *NVE* runs, the pressure and temperature of the systems were monitored to ensure that they corresponded to the desired values.

The nine individual equilibrated pure polymer configurations (three *M*_w_ systems at three different temperatures each) were subsequently loaded with CO_2_ at three different concentrations for each temperature (reported in Table 1); therefore, the total number of individual gas–polymer systems is 27. Each gas–polymer system underwent a similar sequence of equilibration steps: energy minimization, 5 ns *NVT* run followed by a series of 20 ns *N*_1_*N*_2_*PT* in the mixture case to compute the equilibrium pressure corresponding to each value of concentration chosen. This is accomplished with an iterative procedure described in the following paragraphs. Once this step was completed, the system was further equilibrated for 20 ns in the *NVT* ensemble, and finally 50–100 ns production runs were performed in the *NVE* ensemble.

### Sorption Isotherms Calculation

The concentration values used to load the polystyrene models with CO_2_ were chosen from the analysis of experimental data of CO_2_ sorption isotherms at different temperatures^23^ and by extrapolating the behavior to higher temperatures of interest using the Sanchez–Lacombe (SL) equation of state.^50^ A reparametrization of the equation of state was performed, since SL parameter sets reported in the literature for aPS were unable to represent the effects of *M*_w_. Details of the procedure adopted in the regression of SL parameters are reported in the next section and in the Supporting Information.

To achieve consistency between gas concentration and equilibrium pressure, an iterative scheme was applied consisting of the following steps.^47^ At first, a 20 ns *N*_1_*N*_2_*PT* run was performed by using a guess value for the target pressure. The initial guess values were chosen as the equilibrium pressures calculated with the Sanchez–Lacombe equation of state corresponding to each concentration value. Afterward, Widom test particle insertions^41^ were performed in well-equilibrated trajectories obtained from simulations in the *NPT* ensemble to compute the excess chemical potential of CO_2_ (μ_CO_2__^ex^) in the system via the relation^61^
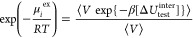
1where β = 1/*k*_B_T, *k*_B_ is the Boltzmann constant, *T* is the temperature, and Δ*U*_test_^inter^ is the change in the intermolecular energy of the system brought by the insertion of the additional molecule (i.e., the potential energy of interaction between the test molecule and the other molecules of the system). The last half of the well-equilibrated part of the trajectory is used (10 ns), which corresponds to 2000 frames. In each frame, 1000 insertions are performed, corresponding to 2 × 10^6^ insertions in total. Insertion positions and the orientation of the molecule to be inserted were chosen at random from a pure CO_2_ trajectory, sampling the configuration space of the pure gas at the same temperature. CO_2_ fugacity in the gas–polymer system was obtained from the chemical potential as follows:^26^
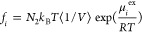
2*N*_2_ is the number of CO_2_ molecules in the polymer matrix, ⟨1/*V*⟩ is the average inverse volume of the system under *N*_1_*N*_2_*PT* conditions, *k*_B_ is the Boltzmann constant, and *T* is the temperature of the simulation. The phase equilibrium condition implies the equality of fugacity of CO_2_ in the gas phase and dissolved in the polymer. Therefore, the total pressure corresponding to the CO_2_ fugacity was calculated by using the Peng–Robinson equation of state.^62^ This new value of the pressure was used to perform a new 20 ns *N*_1_*N*_2_*PT* run. This procedure was repeated until the pressure value calculated from the EoS based on the fugacity extracted from Widom insertions in the configurations of the MD trajectory converged between subsequent iterations. Convergence was reached after three iterations at maximum. By performing Widom insertions on pure polymer configurations, we obtained the excess chemical potential at infinite dilution, from which the Henry’s law constant can be calculated by using the following mass-fraction-based relation:
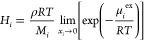
3In eq 3, ρ is the mass density of the pure polymer system and *M*_*i*_ is the molar mass of CO_2_.

### Sanchez–Lacombe EoS Parameters Regression

Equation-of-state parameters for pure polymers are usually obtained from the best fit to pressure–volume–temperature (*pVT*) data sets above the glass transition, as EoS models do not apply to the nonequilibrium state of glassy polymers. To perform an analysis of the *M*_w_ effects on various properties with the Sanchez–Lacombe EoS, the ability of existing parameter sets to account for the difference in *pVT* properties at different *M*_w_ was tested by using the experimental measurements of Zoller and Walsh^63^ as targets. Three different parameter sets were tested,^20,21,64^ but they were able to represent correctly only the properties of high-*M*_w_ polystyrene and failed to account for the *M*_w_ effect on *pVT* properties. For this reason, new parameter sets valid also for the low *M*_w_ range were determined from a best fit to the data reported by Zoller and Walsh.^63^ The maximum deviation between experimental data and EoS results was 1.1% at 110000 g/mol, 1.0% at 34500 and 9000 g/mol, and 2.5% at 910 g/mol. The average deviation was 0.4% in all cases. A functional dependence of the parameters on *M*_w_ was obtained, as detailed in the Supporting Information, that enabled the calculation of the parameters corresponding to the *M*_w_ under study, which are reported in Table 2.

**Table 2:** Sanchez–Lacombe EoS Parameter Sets for CO_2_^65^ and aPS at Different *M*_w_^a^

*M*_w_ (g/mol)	T*(K)	P* (MPa)	ρ* (g/cm^3^)
2100	744 ± 10	390 ± 29	1.077 ± 0.004
5200	748 ± 10	381 ± 27	1.090 ± 0.002
31000	750 ± 10	371 ± 24	1.098 ± 0.003
CO_2_^65^	300	630	1.515

a 95% confidence intervals accounting for coupling between the parameters were estimated by using a bootstrap method.^66^

## Results and Discussion

### Volumetric Properties and Chain Dimensions

**Figure 1:**
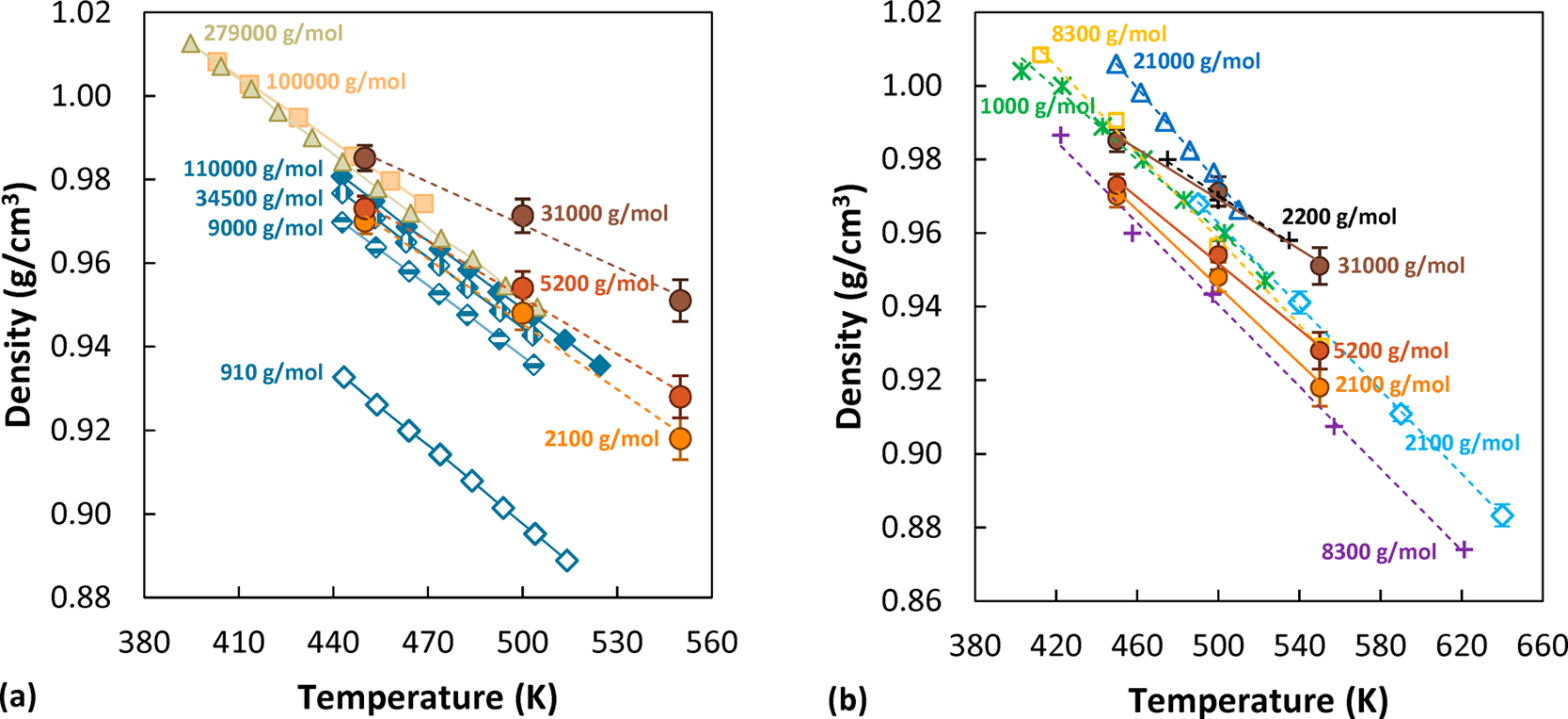
Density of the simulated aPS systems (circles). Brown: 31000 g/mol. Red: 5200 g/mol. Orange: 2100 g/mol. (a) Comparison with experimental values: yellow squares from ref (67), green triangles from ref (68), and blue diamonds from ref (63). (b) Comparison with simulated values: dark blue triangles from ref (44) (AA), light orange squares from ref (69) (UA), green asterisks from ref (70) (AA), light blue diamonds from ref (36) (AA), purple pluses from ref (71) (UA), and black pluses from ref (33) (AA).

The ability to predict volumetric properties is an important prerequisite of the chosen model for a reliable prediction of sorption and diffusion. Average density values were computed for each pure polymer system from the second half of the *NPT* trajectory, over 10 ns. The results obtained for the pure polymer systems are compared in Figure 1a against experimental data at different *M*_w_ from several studies and in Figure 1b against results of other simulation studies.

As expected, the density of the systems decreases with temperature and increases with *M*_w_. The simulations tend to overestimate the experimental behavior: a 2% deviation at 450 K and 3% at 500 K are present between our results at 31000 g/mol and the curve at 34500 g/mol reported by Zoller et al.^63^ Similar results for density were obtained by different authors in molecular modeling studies of polystyrene, with both all atom (AA) and united atom (UA) representations, as can be seen by comparing the data reported in Figure 1b. In most of the cases, as in the present work, low-*M*_w_ curves display values closer to the experimental ones of a much higher *M*_w_; therefore, limited success in capturing *M*_w_ effects on the density seems to be a common issue across different molecular representations. A possible explanation for this density overestimation could be that in the force field adopted the nonbonded interactions are optimized to reproduce the density of a high-*M*_w_ material in simulations of moderately long chains.

The temperature dependence of the density was assessed by calculating the thermal expansion coefficients at atmospheric pressure for the pure polymer systems. The results obtained are 3.53 × 10^–4^ K^–1^ for the high-*M*_w_ system, 4.74 × 10^–4^ K^–1^ for the intermediate-*M*_w_ system, and 5.52 × 10^–4^ K^–1^ for the low-*M*_w_ system. The thermal expansion coefficient for the higher *M*_w_ simulated is of the same order of magnitude as the experimental values, approximately by a factor of 2 lower. The data of Zoller et al.^63^ indicate that the thermal expansion coefficient increases with decreasing *M*_w_, which is consistent with the results obtained in this work. In Table S5 the values calculated from all the data reported in Figure 1 are listed for comparison.

Fox and Flory reported density data at 490 K and different molecular weights for aPS melts, finding a linear relationship between specific volume and inverse *M*_w_.^72^ The simulated values are thus reported in the same fashion in Figure S3, showing a reasonable agreement with linearity, considering that only three data points are available for analysis. Furthermore, comparing the experimental curve at 490 K and the simulation data at 500 K, one can see that the slope of the line interpolating the simulation results is similar to the experimental one, although the absolute values of the simulated specific volume are lower. The best agreement is observed at the intermediate *M*_w_ (5200 g/mol), whereas the lower *M*_w_ and the higher *M*_w_ are both denser than experiment.

**Figure 2:**
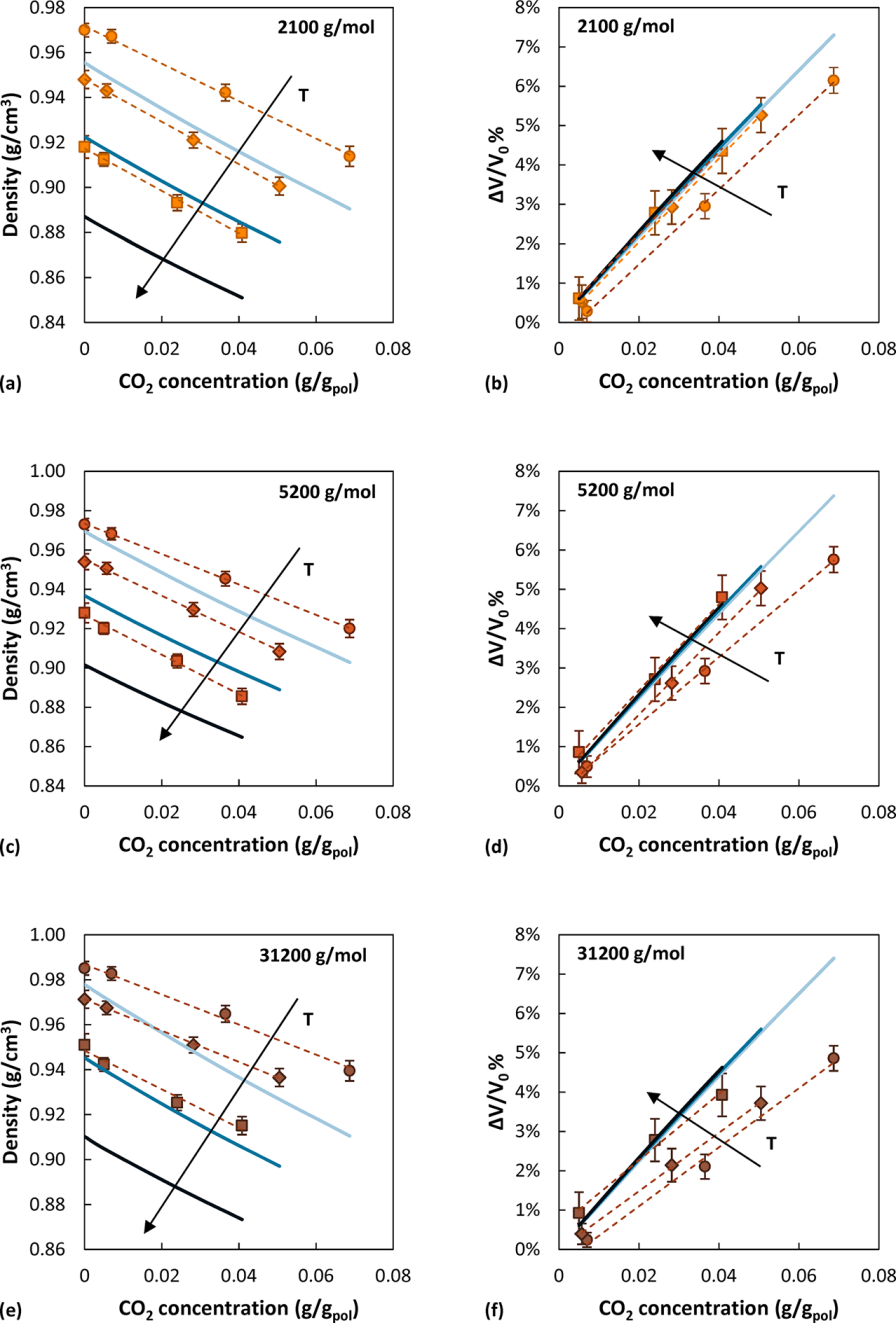
Comparison of simulation and EoS calculations of aPS density and relative aPS volume dilation as a function of CO_2_ concentration. Circles represent data at 450 K, diamonds at 500 K, and squares at 550 K. The *M*_w_ of 2100 g/mol is depicted in orange, 5200 g/mol in red, and 31000 g/mol in brown. Dashed lines are linear interpolations to guide the eye. For some of the cases error bars are smaller than the symbol size. Solid lines are calculated with the SL EoS: light blue at 450 K, blue at 500 K, and dark blue at 550 K.

**Figure 3:**
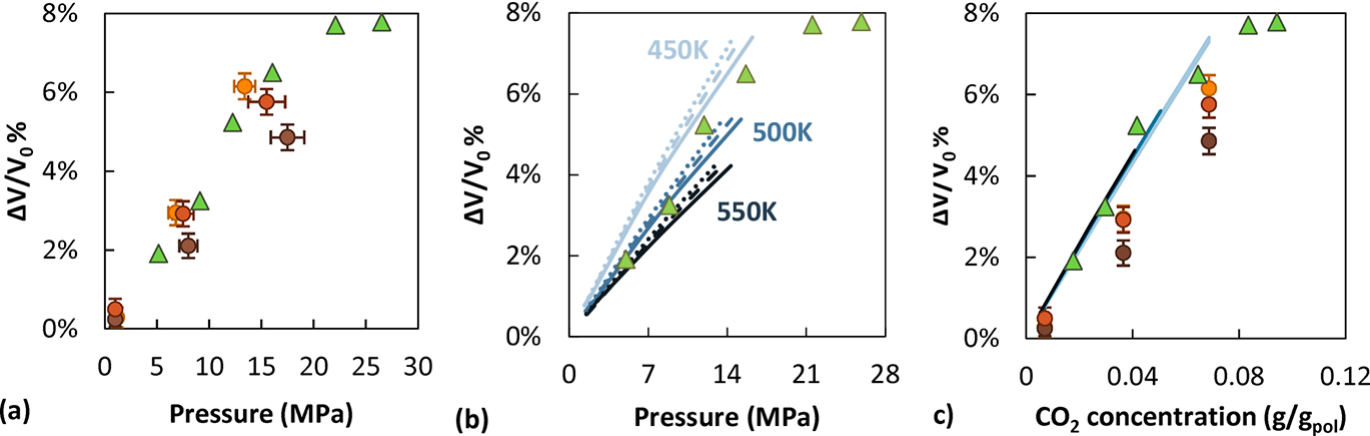
Comparison between CO_2_-induced swelling in aPS for the simulated systems at 450 K (orange represents *M*_w_ of 2100 g/mol, red 5200 g/mol, and brown 31000 g/mol), the experimental measurements of Pantoula et al.^15^ (green triangles, 405 K, *M*_w_ = 230000 g/mol), and SL EoS predictions. Light blue represents data at 450 K, blue at 500 K, and dark blue at 550 K. In (b) solid lines represent 31000 g/mol, dashed lines 5200 g/mol, and dotted lines 2100 g/mol. In (c) curves for different *M*_w_ are collapsed onto one another.

In Figure 2, the density of the systems as a function of CO_2_ content is reported. The values are computed as averages over the second half of the last iteration of *N*_1_*N*_2_*PT* trajectories, when the pressure had converged to its equilibrium value. A linear trend is followed, as often observed also experimentally in the case of sorption of light gases in rubbery polymers.^73,74^ The slope of the linear trend is very similar across temperatures and *M*_w_, signifying that systems at different temperatures and *M*_w_ that are exposed to the same CO_2_ concentration dilate to a similar extent. This is even more apparent in calculated swelling curves (percent volume change), reported also in Figure 2. The percent volume dilation of the polymer increases as the pressure and consequently gas concentration increase, as reported experimentally,^75^ with a linear trend in the pressure range investigated. No experimental measurements for the dilation of aPS as a function of CO_2_ concentration at these temperatures were available in the literature; therefore, the results were compared with the predictions of the SL EoS. To perform calculations with the EoS, the CO_2_–aPS binary interaction parameter was determined from the best fit of experimental sorption isotherms at the highest temperatures available and then extrapolated quadratically to the temperatures of the simulations. More details are given in the Supporting Information. The equation of state was parametrized on pressure–volume–temperature experimental data; therefore, it closely reproduces the density of the pure polymer at different molecular weights. As can be seen in Figure 2, the highest difference between EoS and simulation results is observed at 550 K, with an average deviation of 3.4% with respect to the simulated value (1.7% at 450 K and 2.7% at 500 K). While the absolute density values show larger deviations at higher temperatures, the slope of the dilation curves also tends to be lower in the simulations, especially at lower temperatures and higher *M*_w_, as can be observed in Figure 2 by comparing the blue curves corresponding to EoS predictions and the filled symbols referring to simulated results. The same conclusion can be drawn by observing percent volume change curves. In Figure 3a, the simulation results at 450 K are compared to data by Pantoula et al.^15^ at 405 K on a sample of 230000 g/mol *M*_w_. Even though the experimental sample was tested at a lower temperature and had a 2 orders of magnitude higher *M*_w_ than the low- and intermediate-*M*_w_ systems simulated here, the swelling is very similar. In Figure 3b, EoS results at 450−550 K are plotted against measurements at 405 K by Pantoula et al.^15^ and an overestimation of the swelling as obtained from the EoS predictions is observed. However, equation-of-state predictions indicate that the *M*_w_ dependence and the *T* dependence of percent volume swelling are negligible when comparing data at the same sorbed concentration. Remarkably, also the experimental data by Pantoula et al.^15^ fall onto this master curve (Figure 3c). Plotting the simulated values at constant concentration does not yield a generalized trend for the simulated systems, as can be seen also in Figure 2b,d,f. At the highest temperature, data from all *M*_w_ fall into the master curve, while at 500 K the highest *M*_w_ curve deviates from the others. At 450 K, all systems exhibit lower swelling compared to the EoS predictions. Royer et al.^75^ reported the swelling induced by CO_2_ in rubbery poly(dimethylsiloxane) (PDMS) at 30, 50, and 70 °C, finding that swelling increases from 30 to 50 °C but decreases at 70 °C. Interestingly, in their work the curves at different temperatures displayed a certain degree of superposition, especially below 200 atm, similarly to the observations from the present simulations of polystyrene. Royer et al.^75^ observed for PDMS a different slope of the curves of swelling vs pressure at different temperatures, while in this study systems at different temperatures but at fixed *M*_w_ have the same slope. Looking at the *M*_w_ dependence at fixed temperature, we observed that the higher stiffness of the high-*M*_w_ aPS chains makes them less susceptible to CO_2_-induced dilation, and the system exhibits lower swelling compared to the other systems, especially at higher temperature. Royer et al.^75^ reported an analysis of *M*_w_ effects on polymer swelling induced by CO_2_ in PDMS from 95 to 284 kg/mol. They found little effect of *M*_w_ on swelling. However, they investigated samples with *M*_w_ higher than the entanglement *M*_w_ and advised that the *M*_w_ dependence could be more marked at lower *M*_w_.

The isothermal compressibility of the systems was calculated from the volume (*V*) fluctuations during the *NPT* runs by using the following relation:
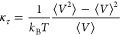
4The results are reported in Figure S5. Even though there is some scattering, a decreasing trend with increasing *M*_w_ and decreasing temperature can be observed. The results are in the range 3 × 10^–10^–1.3 × 10^–9^ Pa^–1^ and compare well with the experimental values, reported to be 5.30 × 10^–10^–1.13 × 10^–9^ Pa^–1^ in the temperature interval 373–593 K.^76^ The agreement is significantly better compared to the result 2.50 × 10^–8^ obtained by Spyriouni et al.^32^ using a CG representation that is very close to the one adopted in the equilibration of the high-*M*_w_ chain system here. They identified as the source of this discrepancy the poor transferability of CG intermolecular interactions to high pressures, as a result of which the CG force field fails to reflect the true compressibility of the material. In this work, the CG representation has been employed only in the equilibration stage, while all production runs were performed by using an AA representation and force field, which do not suffer from the same limitation.

Values for the root-mean-squared radius of gyration, ⟨*R*_g_^2^⟩^1/2^, were obtained for all the systems and are reported in Figure S6 as a function of CO_2_ content. The result obtained for the system of *M*_w_ 2100 g/mol at 500 K is 9.64 ± 0.2 Å, which is in very good agreement with the value of 9.86 ± 0.06 Å obtained with the same all atom model and at the same *M*_w_ by Ndoro at al.^36^ The values obtained are practically independent of temperature, which is in agreement with the experimental measurements reported by Boothroyd et al.^77^ for the radius of gyration of polystyrene melts in the temperature range 393–513 K. Despite having a swelling effect on the system, increasing CO_2_ concentration does not appear to significantly affect the average radius of gyration of the chains at any *M*_w_. By looking at individual chains, the addition of the CO_2_ molecules to the high-*M*_w_ system resulted in a very modest but systematic increase in the radius of gyration of all chains, between 1% and 1.5% with respect to the corresponding values in the absence of CO_2_. In the lower *M*_w_ systems, which are endowed with higher mobility and can rearrange their conformation more easily, individual chains displayed either an increase or a decrease in their radius of gyration with increasing CO_2_ concentration, and on average, these variations canceled out. The *M*_w_ dependence of ⟨*R*_g_^2^⟩^1/2^ is captured very well in comparison to values determined by neutron scattering for monodisperse aPS ranging from 21000 to 1100000 g/mol at 393 K,^78^ as shown in Figure S7.

### Radial Distribution Functions

#### Polystyrene

**Figure 4:**
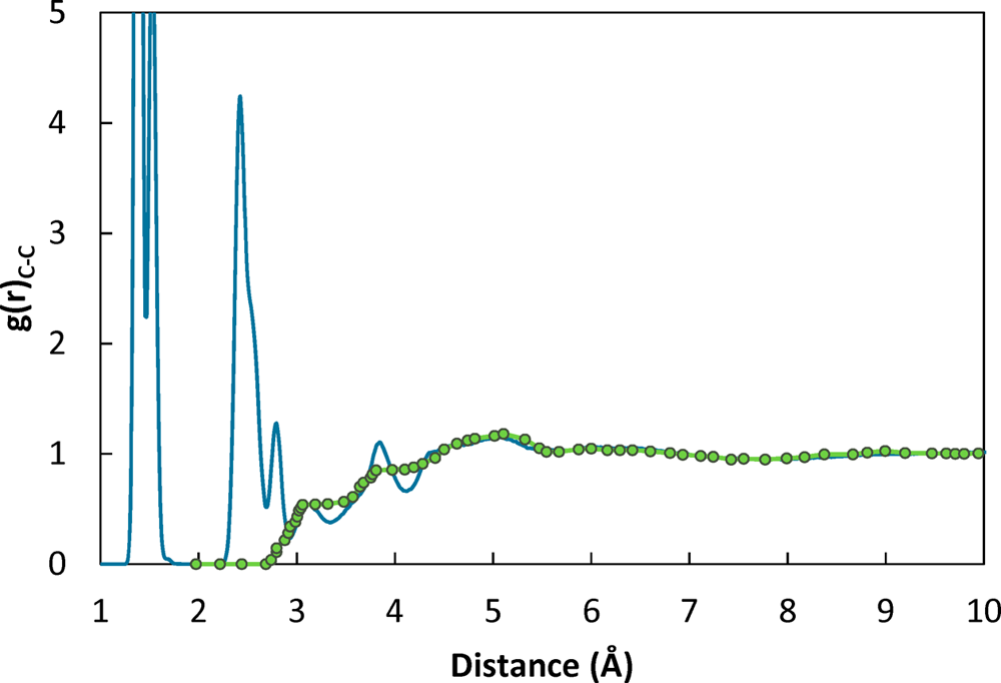
Radial distribution function of all pairs of carbon atoms of the polymer chains. The solid lines represent the simulated system of *M*_w_ 2100 g/mol at 500 K. Green circles are the experimental measurements of Londono et al.^79^ at 323 K for a sample with *M*_w_ 794 g/mol.

**Figure 5:**
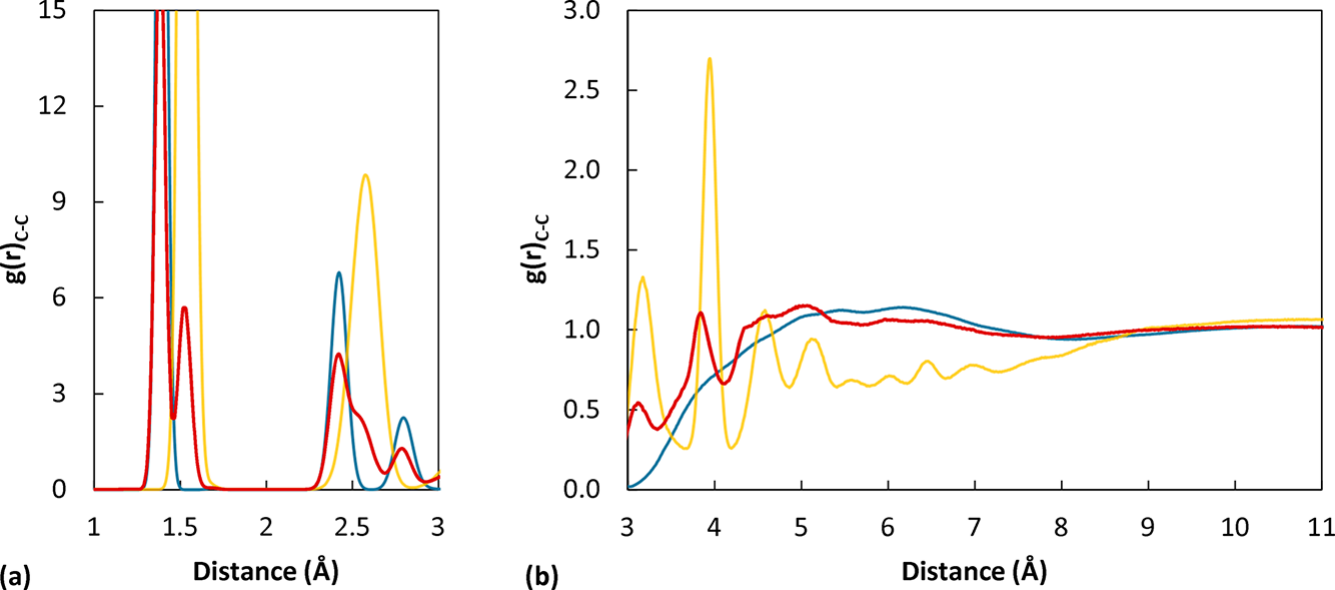
Radial distribution function of pairs of carbon atoms (2100 g/mol, 450 K). Red lines represent the overall correlations of carbon atoms, yellow lines the correlations of backbone carbons, the blue ones the correlations of ring atoms.

Radial distribution functions (RDF, indicated also with *g*(*r*)) provide a quantitative description of molecular packing. They can be readily obtained for different pairs of atoms of the simulated systems.^58^ In the case of macromolecular systems, the short-range features of the curve are related to intramolecular contributions, while at larger distances intermolecular correlations are also present. Figure 4 shows the RDF calculated for all pairs of carbon atoms of the polymer chain. In the Supporting Information the link between the short distance peaks and bonded contributions is shown (Figure S8) as well as the statistical uncertainty associated with this property. Notably, the position and shapes of the features are in good agreement with experimental measurements for the nontrivial intermolecular features, as can be seen in Figure 4, where the RDF of carbon atoms for the simulated system is compared with the experimental one reported by Londono et al.^79^ in which the contributions from first and second bonded neighbors have been excluded. This indicates that the adopted model is capable of representing the local structure of the polymer closely. Peaks at ∼5 Å were interpreted by different authors as containing both interchain contributions and intramolecular correlations,^80^ possibly deriving from phenyl interactions in sequences of monomers with different tacticity.^81^ Peaks located at higher distances, around 6 and 10 Å, were interpreted as originating predominantly from interchain contributions. A magnification of this region of the RDF of the simulated system shows weak features located at these distances as well. By computing the correlations of ring and carbon atoms separately and comparing the results with the overall RDF of carbon atoms (Figure 5), it appears that the features at distances of 5–6 Å are indeed originating predominantly in correlations involving the ring carbons, while around 10 Å the backbone correlations are stronger, as suggested also by experimental findings.^80,81^

**Figure 6:**
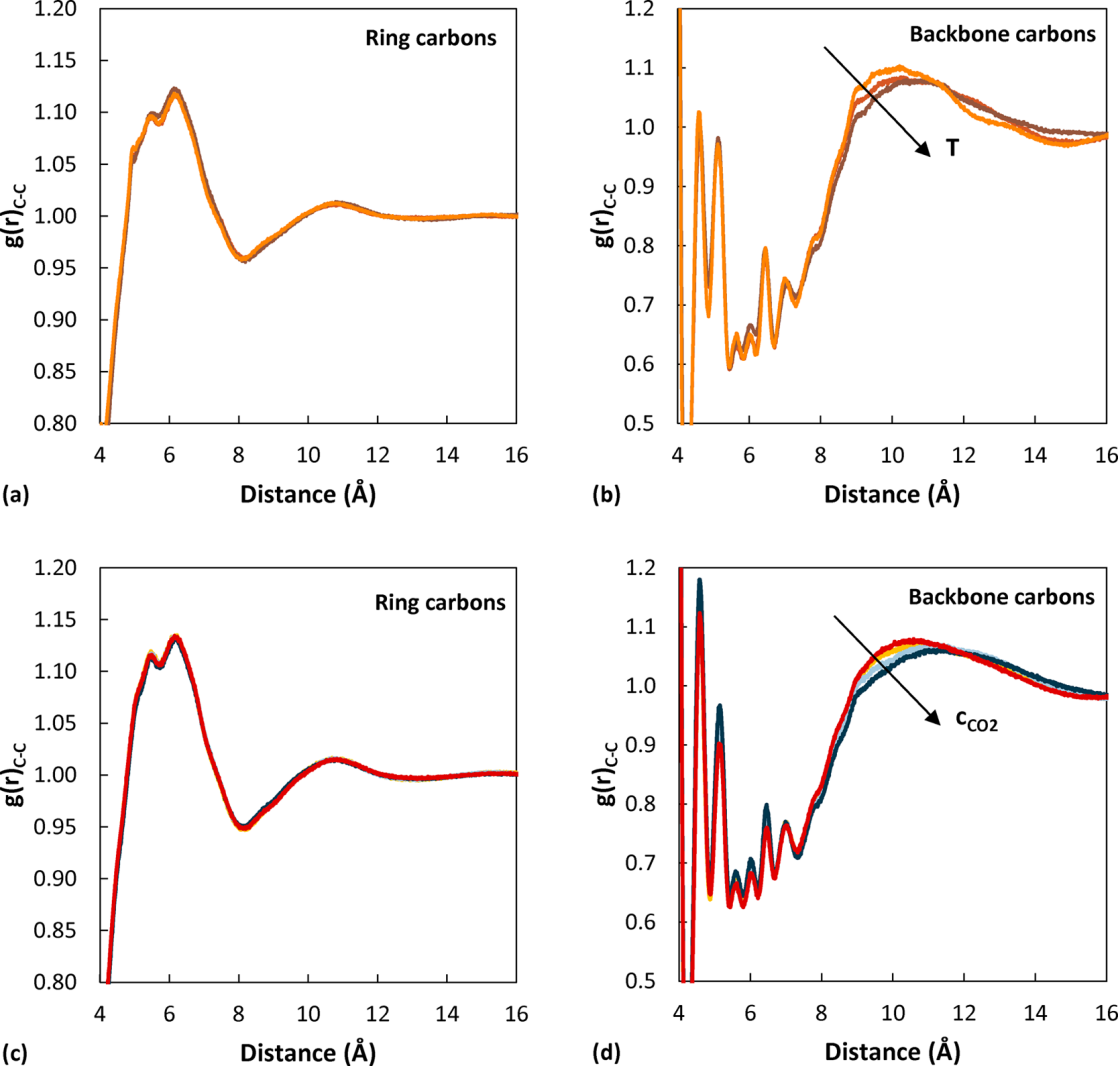
(a, b) Effect of temperature on the peaks of the RDF of pairs of carbon atoms (pure polymer *M*_w_ 2100 g/mol). (a) Carbons on the phenyl rings. (b) Carbons on the backbone. Orange represents 450 K, red 500 K, and brown 550 K. (c, d) Effect of CO_2_ concentration on the peaks of the RDF of pairs of carbon atoms (*M*_w_ 2100 g/mol at 500 K). (c) Carbons on the phenyl rings. (d) Carbons on the backbone. Red represent results for the pure polymer, yellow 5.70 × 10^–3^ g_CO_2__/g_pol_, light blue 2.82 × 10^–2^ g_CO_2__/g_pol_, and blue 5.05 × 10^–2^ g_CO_2__/g_pol_.

The temperature dependence of the RDF was also analyzed. It was found that the first peaks at 1.4 and 2.5 Å, associated with first and second neighbors, do not shift to different distances; they become slightly less intense and broader as the temperature increases. The peaks at higher distance also decrease in intensity as the temperature increases, and especially in the case of the feature at 10 Å, a slight shift toward higher distances is observed. The temperature dependence of all the peaks is shown in Figure S9 for the low-*M*_w_ system, but the same trend was observed at all molecular weights. An analysis of the temperature dependence of the ring and backbone correlations, reported in Figure 6, shows that the strongest effect is displayed in the case of the backbone correlations at around 10 Å. The effect of CO_2_ concentration on the RDF of carbon atoms in aPS is also shown in Figure 6. As CO_2_ concentration increases, backbone carbon correlations show a variation similar to the one observed when temperature is increased: the broad peak located at around 10 Å displays a decrease in intensity and shifts toward higher distances. Comparing this result with the effect of CO_2_ on the radius of gyration, it can be inferred that CO_2_ affects interchain packing more significantly than the average chain dimensions. On the other hand, no effect is detected in the corresponding RDF for the carbon atoms of the rings, even at a close resolution. Opposite to what is observed when increasing temperature, an increase in CO_2_ concentration brings about higher peaks at short distances, in the cases of both ring and backbone carbons. This can be partly seen in Figure 6d and also in Figure S10 for even shorter distances.

#### Polystyrene/CO_2_

**Figure 7:**
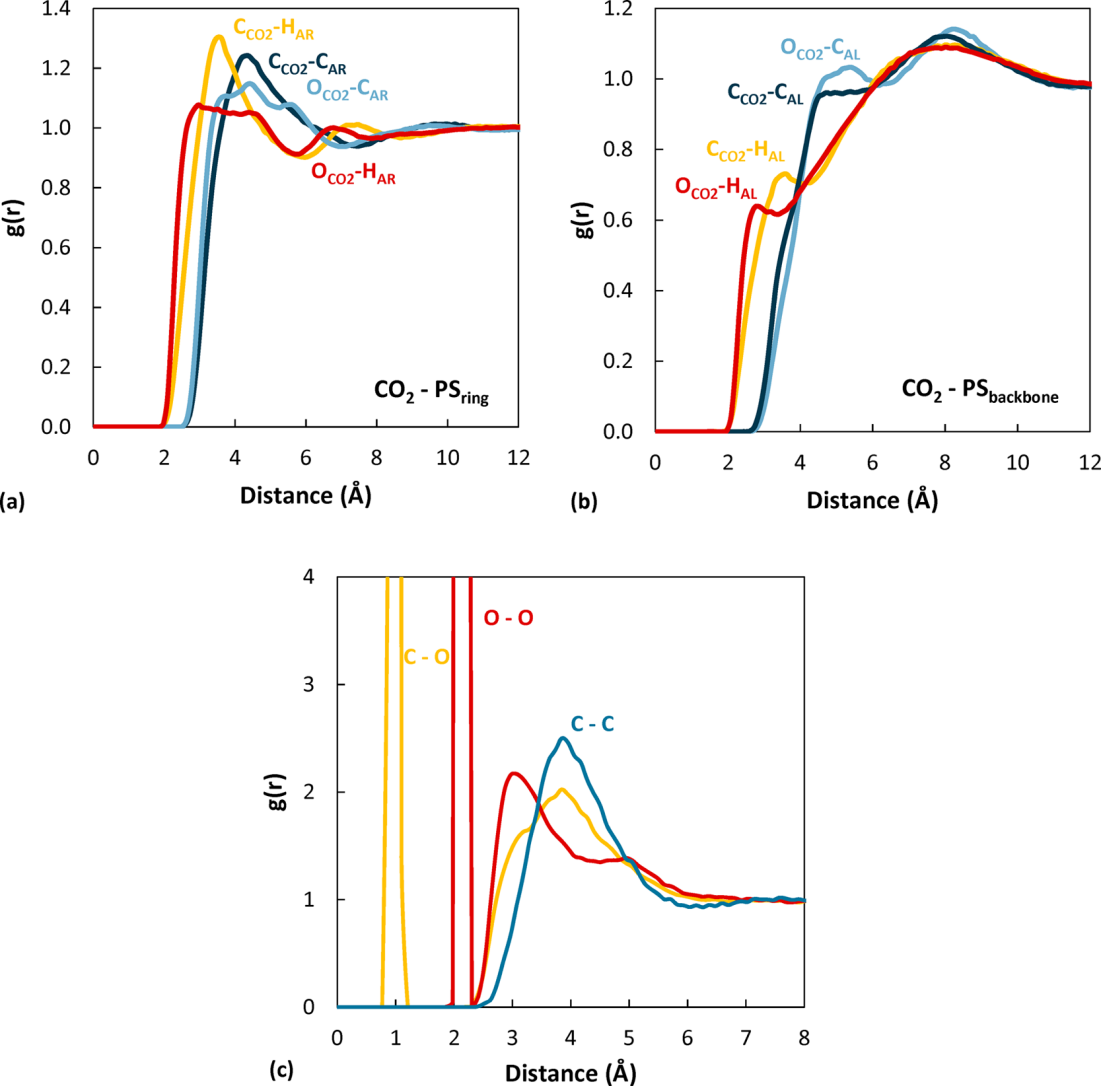
(a, b) Radial distribution functions of pairs of CO_2_–PS atoms in the 2100 g/mol system at 450 K and highest CO_2_ concentration. (c) Radial distribution functions of pairs of CO_2_–CO_2_ atoms in the 2100 g/mol system at 450 K and CO_2_ concentration of 6.87 × 10^–2^ g/g_pol_.

Radial distribution functions between CO_2_ and polystyrene atoms were evaluated as a function of concentration, temperature, and molecular weight of the polymer. Some representative cases are reported in Figure 7 as well as in Figures S11 and S12. Figures 7a and 7b show the correlations of carbon dioxide with polymer atoms on the ring and on the backbone, respectively. In every case, the oxygen atoms of CO_2_ are closer than the carbon atom of CO_2_ to the polymer. Furthermore, CO_2_ is closer to the polymer hydrogens, both on the ring and on the backbone, as expected. Finally, CO_2_ is located closer to the polymer rings than to its backbone. This could be due to the fact that the bulky rings hinder access to the backbone, but it could also originate from the stronger interactions between CO_2_ and the ring due to electrostatic forces. In Figure 7c, the correlations between atoms on different CO_2_ sorbed molecules in the CO_2_/polymer systems are shown. The peaks corresponding to the C–O bond length and intramolecular O–O distance can be recognized at 1.15 and 2.3 Å. Subsequent peaks at 3 and 3.8 Å are associated with the presence of other CO_2_ molecules, indicating that gas molecules are not isolated inside the polymer, even at low concentration. The position of the peaks was invariant with concentration and temperature, but their height decreased at higher temperature.

### X-ray Scattering Patterns

The ability of a molecular model to yield a faithful representation of the material structure can be assessed by comparison with wide-angle X-ray diffraction patterns. The static structure factor *S*(*q*) is related to the scattering intensity: *I*(*q*) ∝ *S*(*q*) + ∑_*i*_*f*_*i*_^2^(*q*), where *q* is the magnitude of the wavevector. It can be calculated from the knowledge of the radial distribution functions *g*(*r*) and atomic form factors *f*_*i*_(*q*):^82,83^
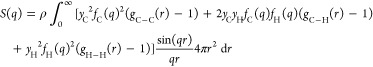
5Features at low *q* values reflect intermolecular correlations in the bulk, whereas peaks at higher *q* values originate in intramolecular correlations; therefore, features at higher distances in the radial distribution functions affect the low *q* region of the structure factor, and vice versa.

#### Polystyrene

For many noncrystalline polymers, the peak at the lowest angle in the scattering curve is the most intense, and it represents interchain correlations. It is also usually found at the same position in scattering patterns of the corresponding monomer. However, polystyrene exhibits a special characteristic: the most intense peak in its wide-angle X-ray scattering (WAXS) pattern is located at 1.4 Å^–1^, but in addition, a diffuse halo at *q* = 0.75 A^–1^ is observed, which, on the contrary, is absent from the scattering pattern of the monomer. Tests performed on mechanically extended samples supported the hypothesis that this peak is associated with spatial correlations between chains, thus being intermolecular in origin.^84^ Moreover, the peak displays an unusual temperature dependence: unlike the peak at 1.4 Å^–1^, which slightly decreases in intensity and shifts toward smaller angles as temperature increases, the peak at 0.75 Å^–1^ increases significantly in intensity with increasing temperature. Further peaks, found at 3.1, 5.6, 9, and 10 Å^–1^,^84^ are negligibly affected by temperature. A decrease in peak intensity with increasing temperature is usually a result from both an increase in thermal disorder and a reduction in electron density due to thermal expansion. The fact that there is no significant effect of temperature on peak shapes or widths is an indication that the polymer expands without any general structural reorganization.^84^

**Figure 8:**
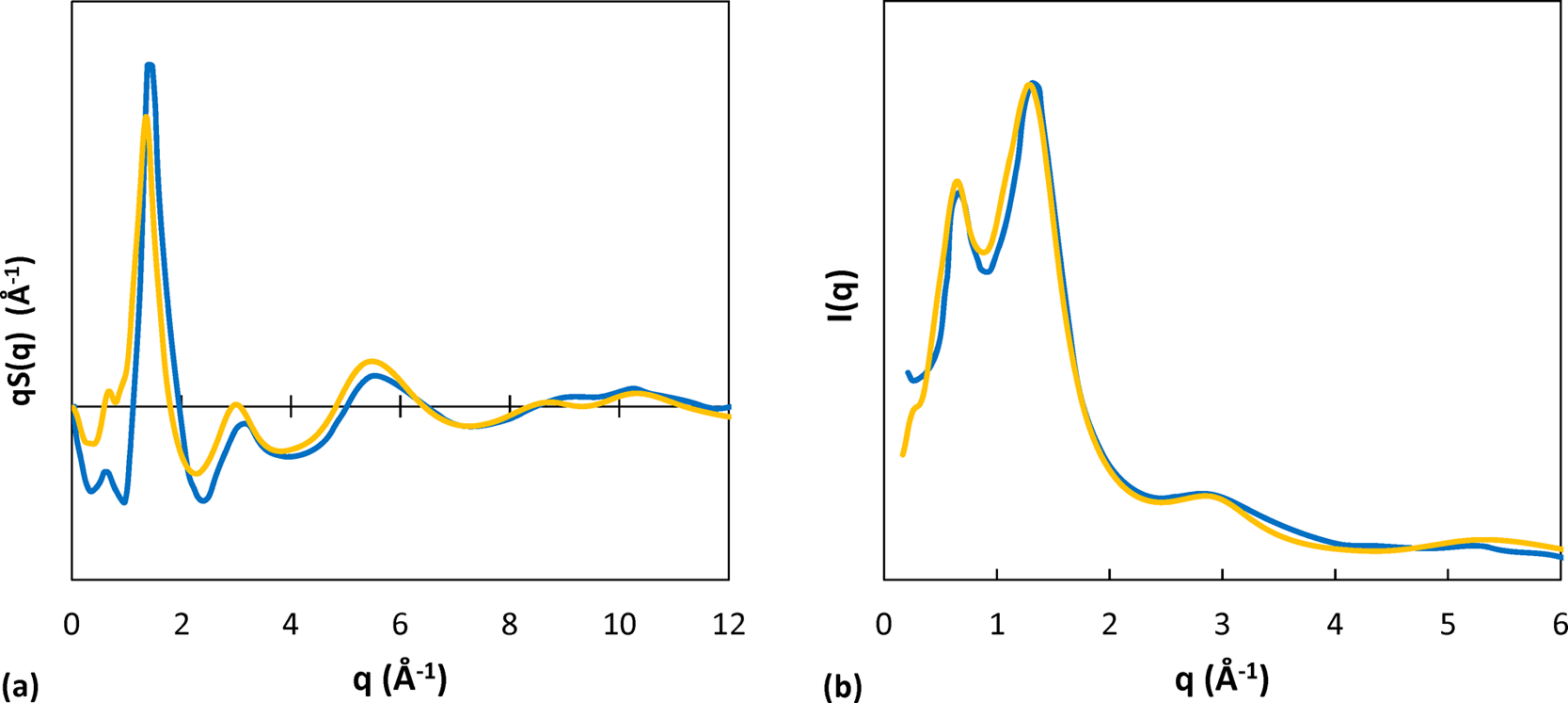
(a) *q*-weighted structure factor of aPS. The blue line is an experimental curve at 293 K;^84^ the yellow one represents the simulation result for the system of 2100 g/mol at 450 K. (b) X-ray scattering intensity of aPS. The blue line is an experimental curve at 523 K;^84^ the yellow one represents the simulation result for the system of 2100 g/mol at 550 K.

The comparison between the experimental structure factor at 293 K^84^ and the simulated one at 450 K (the lowest temperature available in this study for comparison) shows that the positions of the peaks are well predicted (Figure 8a). In addition, the fact that the highest peak at 1.4 Å^–1^ is slightly shifted to lower *q* values at the higher temperature of the simulation is consistent with the experimentally observed temperature dependence of this feature. A scattering intensity curve was available at a higher temperature (523 K),^84^ and it was compared with the simulation results at 550 K, finding remarkable agreement (Figure 8b).

**Figure 9:**
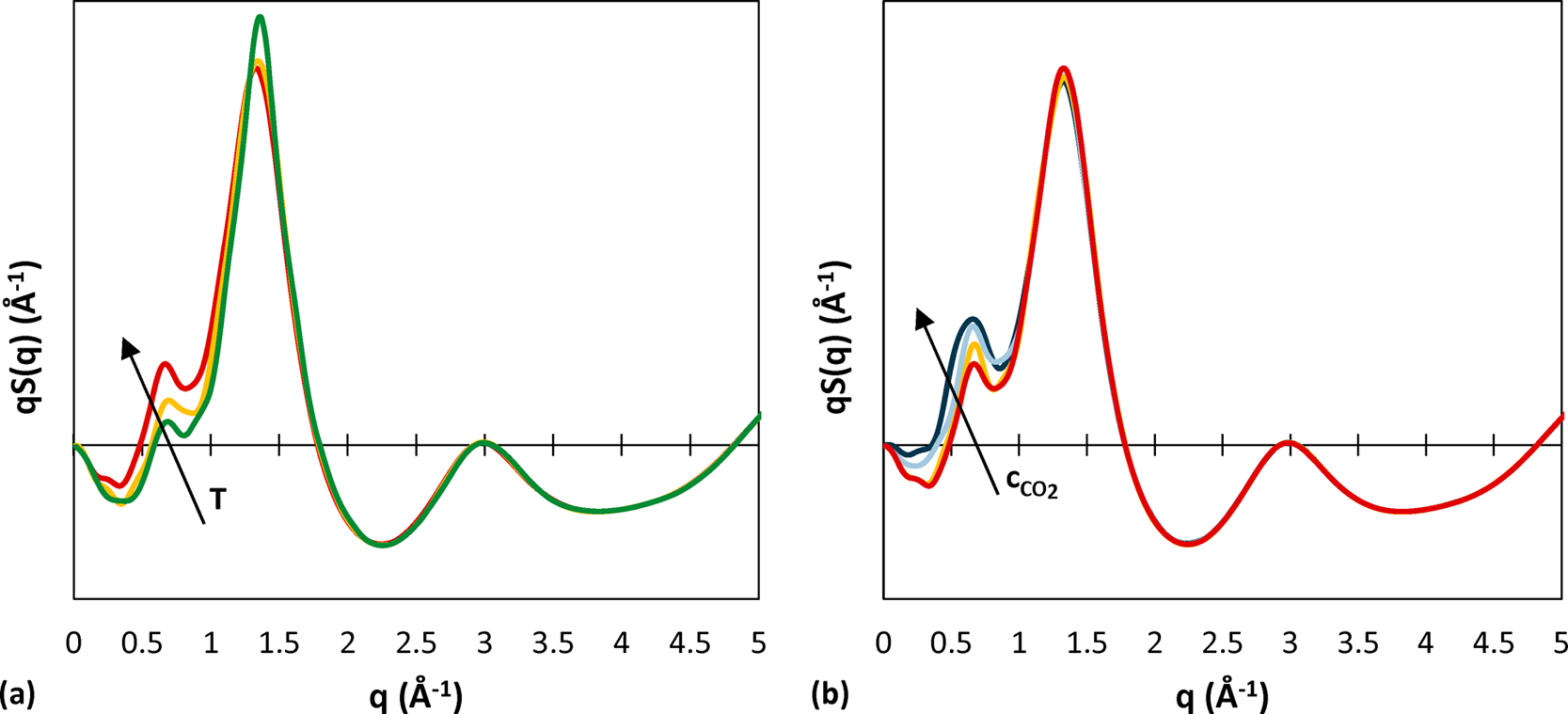
(a) Effect of temperature on the *q*-weighted structure factor of aPS. System: pure polymer *M*_w_ 2100 g/mol. Green represents results at 450 K, yellow at 500 K, and red at 550 K. (b) Effect of CO_2_ concentration on the *q*-weighted structure factor of aPS. System: *M*_w_ 2100 g/mol at 550 K. Red represents results for the pure polymer, yellow 5.00 × 10^–3^ g_CO_2__/g_pol_, light blue 2.40 × 10^–2^ g_CO_2__/g_pol_, and dark blue 4.08 × 10^–2^ g_CO_2__/g_pol_.

The effects of temperature on the structure factor, as obtained from our MD simulations, are displayed in Figure 9. Comparing also with the corresponding radial distribution functions (Figure 5), we attributed the temperature dependence of the first peak mainly to backbone correlations, as the correlation function of the rings does not display a temperature dependence at higher distances. Also, the fact that this peak is not observed in the case of the monomer indicates that it could originate in backbone correlations, which are absent in the case of the monomer. In the range 5–7 Å of the RDF, the dominant contribution is related to ring correlations, and these show indeed a weak temperature dependence, which is assumed to give rise to the second peak and its temperature trend. This interpretation is confirmed also by an analysis of the inter- and intramolecular components of the structure factors of polystyrene obtained from MD trajectories,^85^ where the authors calculated partial structure factors isolating the contributions originating in ring and backbone correlations. This study showed that the peak at 1.4 Å^–1^ arises primarily from phenyl–phenyl correlations, both intra- and intermolecular. While the intermolecular contribution showed a tendency to shift to lower angles with increasing temperature, the intramolecular part was nearly insensitive to temperature, resulting in a weak temperature dependence of this peak. Moreover, the peak at 0.75 Å^–1^ could be ascribed primarily to intermolecular correlations of backbone atoms, which showed the expected decrease in intensity and shift to lower *q* with increasing temperature. However, the superposition of the shift to lower angles of intermolecular phenyl–phenyl and phenyl–backbone peaks gives rise to the anomalous increase with temperature of this peak.

#### Polystyrene/CO_2_

In the presence of CO_2_, one can see that an increase in gas concentration has the same effect on the intensity and position of the first two peaks of the X-ray scattering pattern of aPS as an increase in temperature (Figure 9b). However, as was observed in the analysis of radial distribution functions, an increase in concentration affects only backbone–backbone correlations with a decrease in intensity and shift to higher distances. This is reflected in the shift to lower angles of the 0.75 Å^–1^ peak with increasing CO_2_ concentration.

### Local Dynamics

**Figure 10:**
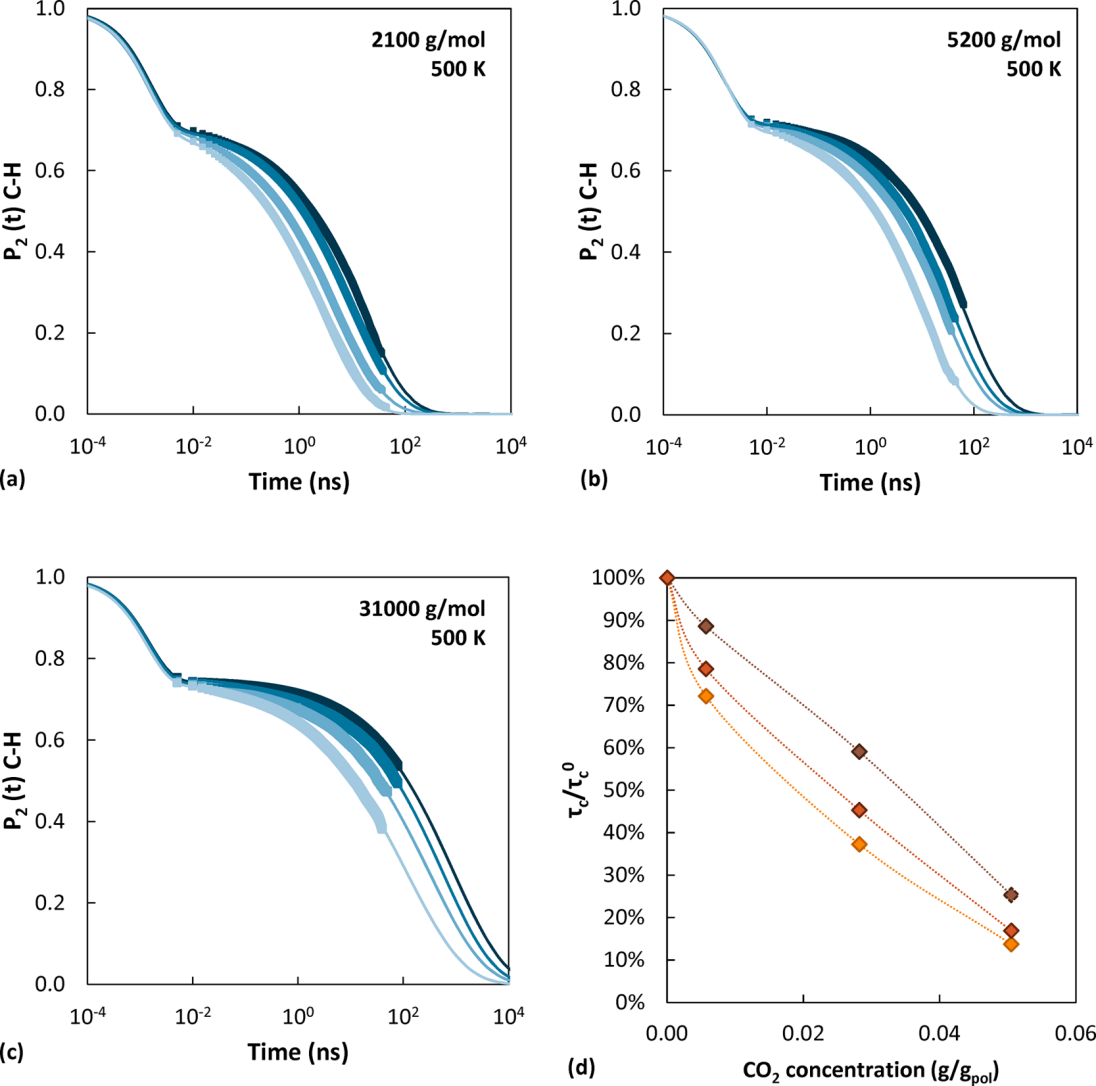
Effect of CO_2_ concentration on the orientational decorrelation of the C–H bonds in pure aPS. The symbols represent simulated data at *M*_w_ (a) 2100 g/mol, (b) 5200 g/mol, and (c) 31000 g/mol at 500 K. Lighter colors indicate higher gas concentration. Solid lines show extrapolation to shorter and longer times with a mKWW function. In (d) the relative decrease in the decorrelation times as a function of CO_2_ concentration with respect to the value of the pure polymer is reported.

The MD trajectories at constant energy were analyzed to extract information about the local dynamics and the effect of temperature, *M*_w_, and gas concentration on the motion of various polymer segments. In the case of polystyrene, the vectors characterizing the orientation of the phenyl ring and the orientation of the C–H bonds are of interest since their decorrelation rates can be compared directly to dielectric spectroscopy (DS) results and NMR measurements. The orientational decorrelation with time is analyzed by considering ensemble-averaged Legendre polynomials of order *k* (*P*_*k*_(*t*)) of the inner product of a unit vector *v̂* with itself at times *t*_0_ and *t* = *t*_0_ + Δ*t*: ⟨*v̂*|_*t*_0__·*v̂*|_*t*_0_+Δ*t*_⟩. To compare the simulation results with DS measurements, the first-order Legendre polynomial (*P*_1_(*t*) = ⟨cos θ(*t*)⟩) is computed by considering the vector that begins from the backbone carbon connected to the phenyl ring and ends at the center of mass of the ring considered (C–com_Ring_ vector), since the dipole moments of the monomer are approximately directed along this vector. θ(*t*) is the angle by which the vector has rotated in a time *t* relative to its original position at the time origin *t*_0_. To compare the simulation results with NMR measurements of spin–lattice relaxation of ^2^H nuclei, the second-order Legendre polynomial is computed (*P*_2_(*t*) = ^3^/_2_⟨cos^2^ θ(*t*)⟩ – ^1^/_2_) for C–H bonds, both on the backbone and on the ring, for consistency with the experimental data used for comparison. The decay of the orientational autocorrelation functions is well represented by a modified Kohlrausch–Williams–Watts (mKWW) equation.^86−88^
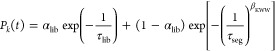
6This function consists of two parts. The first term is associated with a fast exponential decay having amplitude α_lib_. This represents the fast librations of torsion angles around skeletal bonds and the bond stretching and bond angle bending vibrations of skeletal and pendant bonds near their equilibrium values, which have a characteristic time τ_lib_. It is associated with the first hump displayed by the curves shown in Figure 10. The second term, which describes the long-time trend of *P*_*k*_(*t*), is the decay associated with cooperative torsional transitions in the polymer and is represented by a stretched exponential, where τ_seg_ is the characteristic correlation time and β_KKW_ the stretching exponent. By integrating this curve, the correlation time for segmental motion (segmental relaxation time) τ_c_ can be calculated:
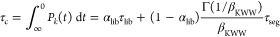
7It is useful to resort to the above fitting procedure for the calculation of the relaxation time, since, in several cases, the orientational autocorrelation function does not decay to zero within the limits of the simulation. As a consequence, the behavior at longer times is estimated by extrapolation with the mKWW function.

In Figure 10, the effect of CO_2_ concentration on the extracted *P*_2_(*t*) and on the calculated relaxation times is shown. Additionally, in Figures S13 and S14 some representative decorrelation functions are displayed to highlight the effects of *T* and *M*_w_ on the segmental dynamics. Analogous trends were obtained also for the cases not shown. The short-time features, associated with fast librations of bonds and angles around their equilibrium values, are insensitive to changes in temperature, gas concentration, and polymer molecular weight as can be seen from the superposition of the first hump of the *P*_2_(*t*) curves in Figure 10 as well as Figures S13 and S14.

**Figure 11:**
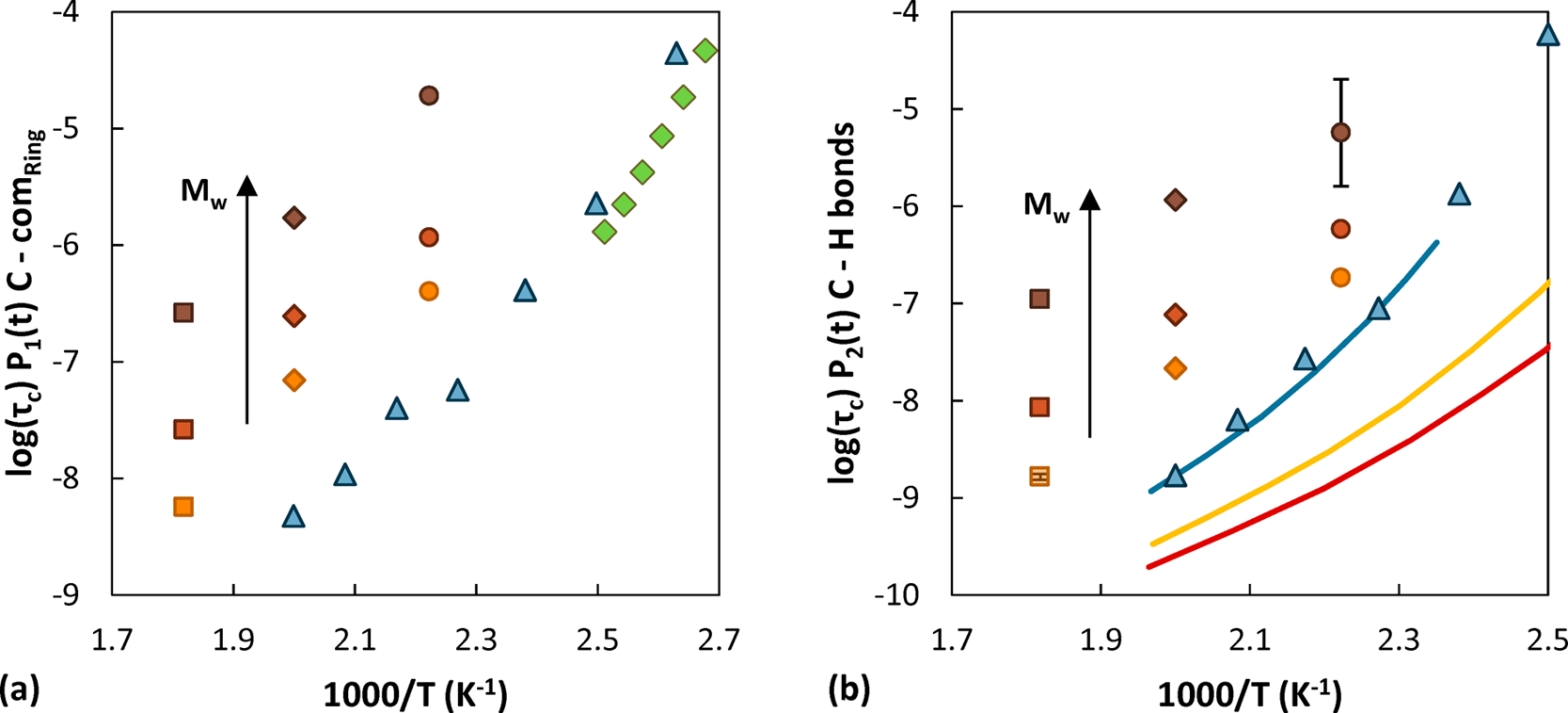
Relaxation times of (a) C–com_Ring_ vectors and (b) C–H bonds. Circles represent data at 450 K, diamonds at 500 K, and squares at 550 K. The molecular weight of 2100 g/mol is depicted in orange, 5200 g/mol in red, and 31000 g/mol in brown. In (a) green diamonds represent experimental data from Pschorn et al.^91^ In (b) lines are NMR measurements by He et al.:^33^ blue corresponds to a sample of 10900 g/mol, yellow to 2100 g/mol, and red to 1600 g/mol. In both (a) and (b) blue triangles are simulation results from the work of Vogiatzis and Theodorou for *M*_w_ = 152000 g/mol.^35^ In (b) error bars are reported for the low-*M*_w_ system at the high-*T* case (smaller than the symbol size) and the high-*M*_w_ system at the low-*T* case.

All effects of temperature, gas concentration, and polymer molecular weight are manifested in the long-time decay associated with cooperative motions. The temperature dependence of the orientational decorrelation followed an Arrhenius behavior over the temperature range considered here, as can be observed clearly in Figure 11, where the logarithm of the relaxation times of the C–H bonds is plotted against the inverse temperature and a linear trend is followed. The same was observed for the C–com_Ring_ vector. Concerning the *M*_w_ dependence, it can be seen in Figure S14 that an increase in molecular weight of the polymer causes the segmental dynamics to become slower. Experimentally, it was observed that in the high *M*_w_ range this dependence asymptotically vanishes. However, in the *M*_w_ range of the simulated systems, an effect of *M*_w_ on the local dynamics was experimentally documented^33^ as well.

Increasing CO_2_ concentration systematically enhances the local dynamics, as can be seen in Figure 10. Systems with higher CO_2_ concentration are less dense, and this promotes the mobility of the polymer. At the same time, a higher gas concentration corresponds to a higher pressure, which would act in the opposite way, slowing the polymer dynamics. However, this effect is overcome by the higher mobility induced by the presence of the penetrant, resulting in shorter decorrelation times at higher concentrations. To compare the relative strength of this effect in the different systems, the relative decrease in the decorrelation times as a function of CO_2_ concentration with respect to the value of the pure polymer was calculated. This is reported in Figure 10d, and it shows that the effect is more pronounced in the case of the lower *M*_w_ system.

The parameters obtained by fitting the mKWW function to the orientational autocorrelation of C–com_R_ vectors for all the systems are reported in Table S6, while Table S7 contains the parameters obtained for the C–H bonds. By analyzing the values of the parameters extracted from fitting the orientational decorrelation function, we observed trends in good agreement with experimental evidence. In the case of the C–H bond, the weighted average of backbone and phenyl bonds is considered. However, a separate analysis of the two contributions showed that bonds on the ring are endowed with faster dynamics than those on the backbone, as was evidenced also experimentally^33^ with relaxation times 1.5–3 times higher, which is consistent also with the results obtained in other simulations.^89^ In the reorientation of both phenyl rings and C–H bonds, the values of α_lib_ obtained are fairly independent of concentration and *M*_w_, while they display a weak decreasing trend with decreasing temperature. The same trend with temperature was obtained also in the work of Vogiatzis and Theodorou,^35^ who simulated the local dynamics of aPS melts, both pure and in the presence of fullerene nanoparticles, using a united-atom representation. In the case of the phenyl rings, very similar values, ranging from 0.03 to 0.05 were obtained for α_lib_, while for the C–H bond, the values obtained here are 3 times higher than those calculated by Vogiatzis and Theodorou.^35^ The difference in the obtained values can be attributed to differences in the force field and the molecular weight of the systems studied. Comparing with the fitting parameters retrieved from the experimental curves measured by He et al.,^33^ one can see that similar values to the ones of the present work are obtained for α_lib_ (on average 0.22 in their work for a 2200 g/mol sample at different temperatures) and also for β (0.45) concerning the relaxation of C–H bonds. On the contrary, the values of τ_seg_ extracted from our simulations are orders of magnitude higher than the experimental ones. In the case of the C–com_Ring_ vector, the obtained values of 0.5–0.6 for the stretching exponent are consistent with those determined by dielectric spectroscopy measurements.^89^ In the case of β there is no apparent dependence on concentration, *T* or *M*_w_. The small variation of α_lib_ and β with temperature is a consequence of the invariance of the shape of the decorrelation curve with *T*. Low values of the stretching exponent, below unity, are indicative of a high degree of cooperativity in the reorientational motion in the melt,^90^ and indeed the values obtained for all cases are of the order of 0.5–0.6. τ_seg_ displays a clear exponential decay trend with increasing concentration for both vectors analyzed at all temperatures and *M*_w_.

**Figure 12:**
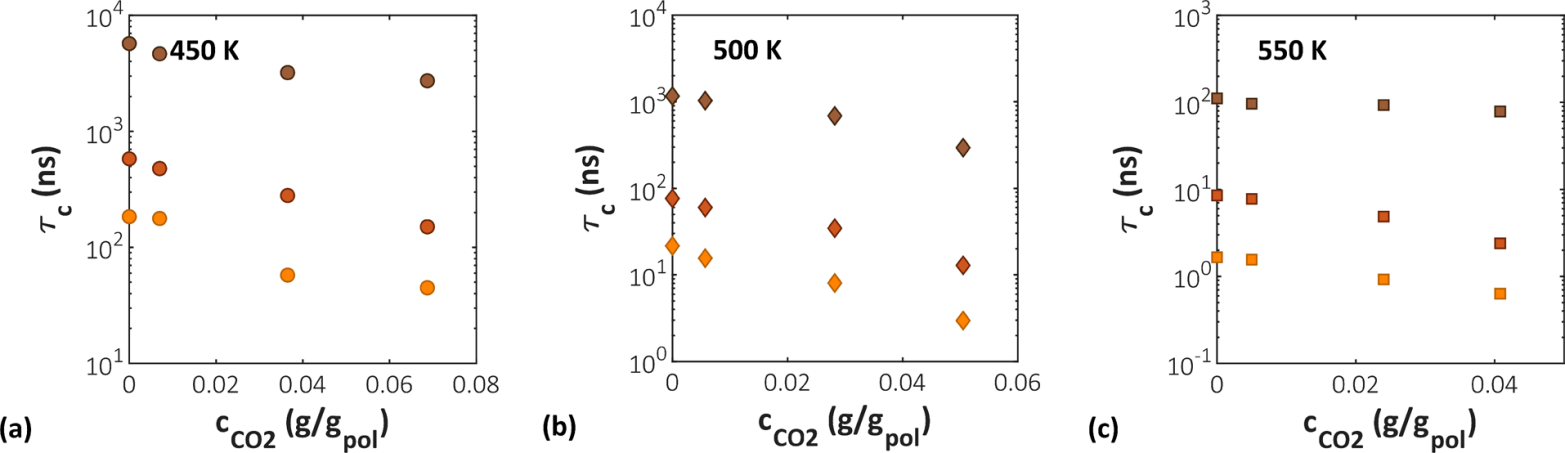
Relaxation times of the C–H bonds of aPS as a function of CO_2_ concentration at different temperatures and molecular weights. Circles represent data at 450 K, diamonds at 500 K, and squares at 550 K. A molecular weight of 2100 g/mol is depicted in orange, 5200 g/mol in red, and 31000 g/mol in brown.

The temperature and molecular weight dependence of the relaxation times were compared with the experimental measurements performed by He et al.^33^ and with the simulation results of Vogiatzis and Theodorou^35^ obtained from a united atom model, which are in close agreement with experimental results and therefore were considered a reliable extrapolation to higher temperatures, where experimental data were not available. The result of the comparison is shown in Figure 11. The decorrelation times obtained in this work are orders of magnitude higher than expected. The temperature trend, however, is better captured. To make a closer comparison, in Figure S15 the experimental results were arbitrarily shifted to superimpose the curve at 2100 g/mol with the simulation results at the same *M*_w_. It can be noted that the three simulation sets display a similar temperature dependence; however, compared to the experimental data, the temperature dependence for the low molecular weights simulated is captured better than in the high-*M*_w_ case. The relaxation times show an exponential decrease with increasing CO_2_ concentration at all temperatures and *M*_w_, as can be seen in Figure 12. Indicative error bars associated with the relaxation times were determined for representative cases and are reported in Figure 11b. The system at low molecular weight and high temperature (bottom left corner of the plot) and the one at high molecular weight and low temperature (upper right corner of the plot) were considered, as they constitute the lower and upper bounds of the uncertainty associated with this property. For both cases the statistical uncertainty was determined by splitting the trajectory in three parts and evaluating the standard deviation of the mean relaxation time determined separately in each subdivision. In the first case, complete decorrelation was observed during the simulation, and the error bar obtained is smaller than the symbol size. In the second case, only partial decorrelation was achieved during the simulation; therefore, the calculation of relaxation times relies on extrapolation with the mKWW function, which introduces a higher degree of uncertainty in the result.

A possible explanation of the slower dynamics exhibited in the simulation is that the adopted all-atom force field does not reproduce correctly the conformational energy barriers of aPS. However, the relative height of the energy barriers for various bonds seemed to be represented correctly, yielding the following ordering concerning the rapidity of orientational decorrelation, from slower to faster: backbone C–C bond, phenyl ring orientation, backbone C–H bond, phenyl C–C bond, average C–H bonds, and C–H bonds on the ring. The same ordering was obtained in other simulation works^89^ and also confirmed experimentally for the vectors that could be probed. Another effect related to the slower dynamics observed in the simulations could be the higher density compared to the experimental values for the corresponding molecular weight. However, pure PS systems with comparable density, which were simulated by using a different interaction potential, yielded more realistic relaxation times.^33^

**Figure 13:**
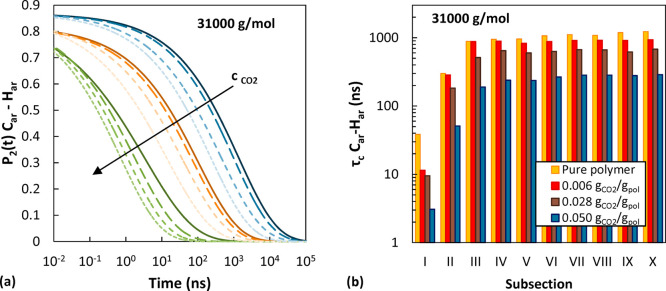
Effect of CO_2_ concentration on the reorientational decorrelation of C_ar_–H_ar_ bonds in different chain subsections for the case of 31000 g/mol at 500 K. In plot (a) green represents the chain end, orange is the second repeating unit starting from the chain end, and blue is the central section of the chain. Solid lines represent the pure polymer case; lighter shades and shorter dashes represent higher CO_2_ concentration. In plot (b) the relaxation time of C_ar_–H_ar_ bonds in each chain subsection is reported as a function of CO_2_ concentration.

The plasticizing effect of CO_2_ was probed at different positions along the chain to assess if it affected more pronouncedly the segments near the chain ends compared to central parts and how this effect is manifested at different molecular weights. As a test case, the second-order Legendre polynomial of the C–H bonds on the rings (C_ar_–H_ar_) was considered at 500 K. The chains were split into 10 subsections from the chain ends up to the chain center, and the orientational decorrelation of the bonds belonging to each subsection was evaluated as a function of CO_2_ concentration. For the low-*M*_w_ case each subsection corresponds to two repeating units located symmetrically with respect to the center of the chain. Table S8 reports how the repeating units were divided for each *M*_w_. For the higher *M*_w_ cases, the bonds belonging to different repeating units grouped in the same subsection displayed relaxation times <10% apart. *P*_2_(*t*) functions were fitted with the mKWW function, and relaxation times were calculated. The results for all cases are reported in Table S9. In Figure 13a, the orientational decorrelation of the C_ar_–H_ar_ bonds is shown as a function of increasing CO_2_ concentration: the first repeating unit is displayed in green, the second repeating unit is displayed in orange, and the central portion of the chain is displayed in blue. Figure 13b shows the relaxation times in all of the ten subsections as a function of increasing CO_2_ concentration for the different *M*_w_ of the polymer chains.

Chain-end effects are generally concentrated only to the three terminal subunits considered. The first and second repeating units display significantly higher mobility at all *M*_w_, whereas toward the center of the chain the relaxation times tend to a plateau value. The same trend is observed at all *M*_w_. Increasing CO_2_ concentration in the system does not seem to affect the range of chain end effects. The presence of the gas leads to a systematic acceleration of the dynamics, but to a comparable extent in all subunits considered, not limitedly to the chain ends. If one computes the relative difference in the relaxation times with respect to the pure polymer as a function of the CO_2_ content in each subunit (shown in Figure S17), it emerges that the chain ends experience a moderately higher acceleration compared to central sections of the chain, but the whole chain is affected in a comparable way as CO_2_ concentration is increased. It should be noted that the results for the higher CO_2_ concentration are endowed with higher accuracy because the higher mobility results in a greater decorrelation during the time of the simulation, reducing the uncertainty in the fitting procedure and in the relaxation times calculated.

The decorrelation of the end-to-end vector was also evaluated as a measure of chain dynamics. The results are shown in Figure S18. The dynamics at the chain level is strongly dependent on the molecular weight, significantly more than the local dynamics of bonds. The higher *M*_w_ case does not show appreciable decorrelation in the time of the simulation (100 ns). This is an indication that the long time scales involved in the high-*M*_w_ systems, even in the melt state, may not be properly sampled by MD. The effect of CO_2_ concentration on the dynamics of the polymer not only is a local effect but also affects the overall mobility of the chain. Also in this case, the relatively higher mobility induced by CO_2_ is more pronounced for the low-*M*_w_ system, as can be observed in Figure S18d, where the orientational decorrelation functions of the three systems at the highest CO_2_ concentrations have been normalized by using the corresponding pure polymer curve. This can be related also to the different swelling experienced by the various systems at the same value of CO_2_ concentration: the low-*M*_w_ systems were more swollen, and the effects on the local chain dynamics were enhanced to a greater extent.

### CO_2_ Sorption Isotherms

**Figure 14:**
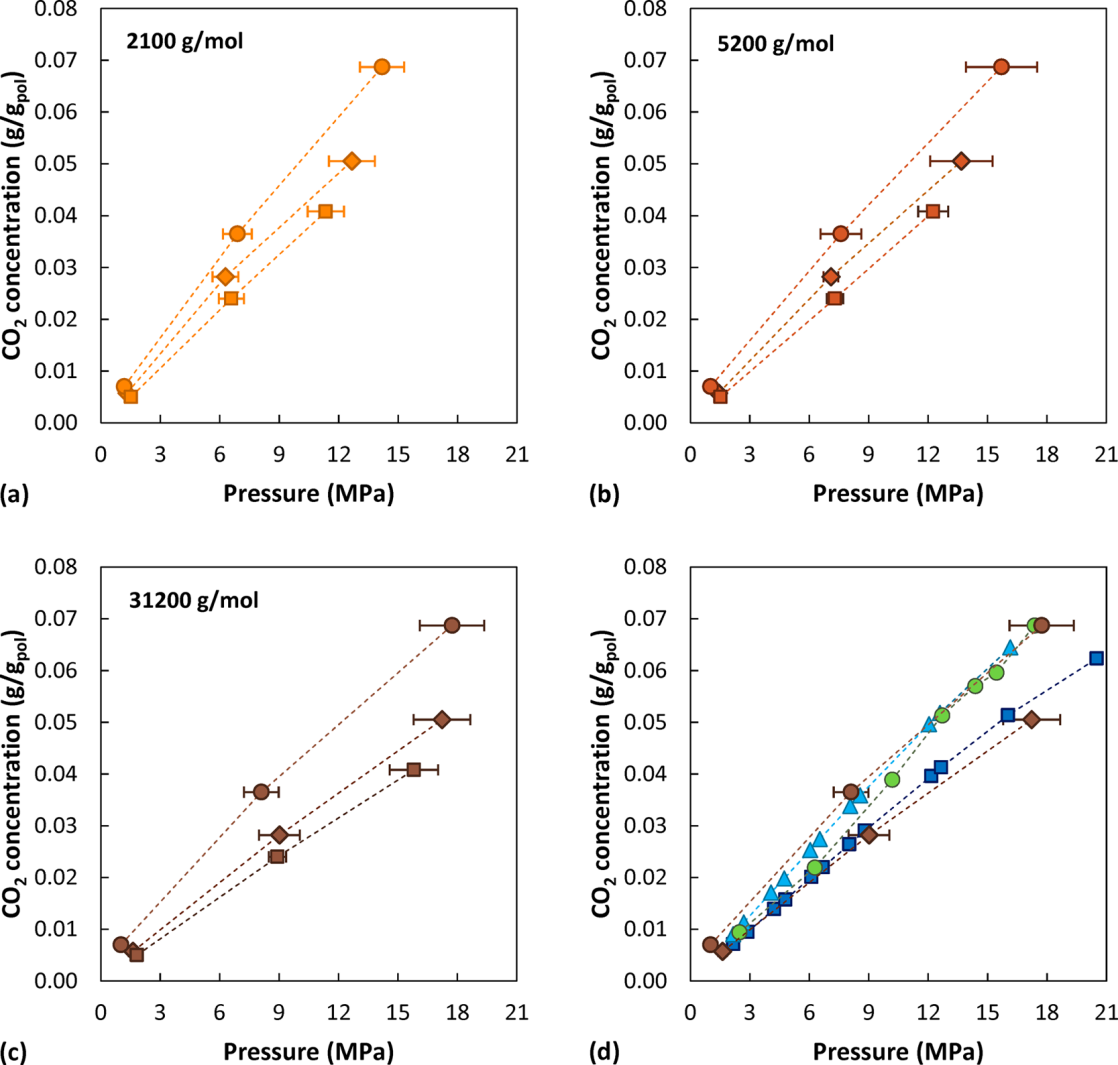
Simulated CO_2_ sorption isotherms in atactic polystyrene at different temperatures and polymer *M*_w_. Circles represent data at 450 K, diamonds at 500 K, and squares at 550 K. A molecular weight of 2100 g/mol is depicted in orange, 5200 g/mol in red, and 31000 g/mol in brown. For data points at low concentrations the error bar is smaller than the symbol size. (d) Comparison between the sorption isotherms simulated at the highest *M*_w_ (31000 g/mol) at 450 K (brown circles) and 500 K (brown diamonds) and the experimental data of Sato et al.^23^ at 423 K (330000 g/mol, blue triangles) and 473 K (330000 g/mol, blue squares). Green circles are data measured by the same group at 453 K^21^ (187000 g/mol).

Using the iterative procedure previously described, we determined the equilibrium pressures corresponding to the value of CO_2_ concentration in each system. The results are reported in Figure 14. A consistent set of results was obtained, with solubility values decreasing with increasing temperature and *M*_w_. The trend of all sorption isotherms is rather linear, as expected for sorption of light gases in rubbery polymers.^74^ The error bars in Figure 14 were obtained with the block average method. The simulated data at the highest *M*_w_ at 450 and 500 K are compared with experimental values of Sato et al.^21,23^ For the tests at 473 and 423 K (blue and light blue symbols) the experimental sample had a *M*_w_ of 330000 g/mol. Green circles are data from for a sample of *M*_w_ of 187000 g/mol at 453 K. As can be seen, the simulated curves overlap with experimental data at a temperature lower by 27 °C, indicating a slight overestimation of the solubility from simulations. However, by taking into account also the variability in experimental data, apparent in the comparison between the green and blue curves in Figure 14, the simulation predictions can be considered very satisfactory.

Toi and Paul^7^ evaluated the effect of *M*_w_ on the sorption isotherms for CO_2_ in glassy polystyrene at different molecular weights ranging from 3600 to 850000 g/mol. Contrary to what was found in this work for the melt state, in the glassy state the extent of sorption increased as the molecular weight increased, a fact that was attributed to the higher fraction of excess free volume present in high-*M*_w_ systems compared to low-*M*_w_ ones, due to the increase in the glass transition temperature as the *M*_w_ increases. In the glass this effect seems to outweigh the increase in free volume associated with a higher number of chain ends, leading to the experimentally observed trend. In the melt state, the unrelaxed excess free volume is not present, and the increasing solubility with decreasing *M*_w_ is associated with a higher number of chain ends that are responsible for a lower density of the matrix.

**Figure 15:**
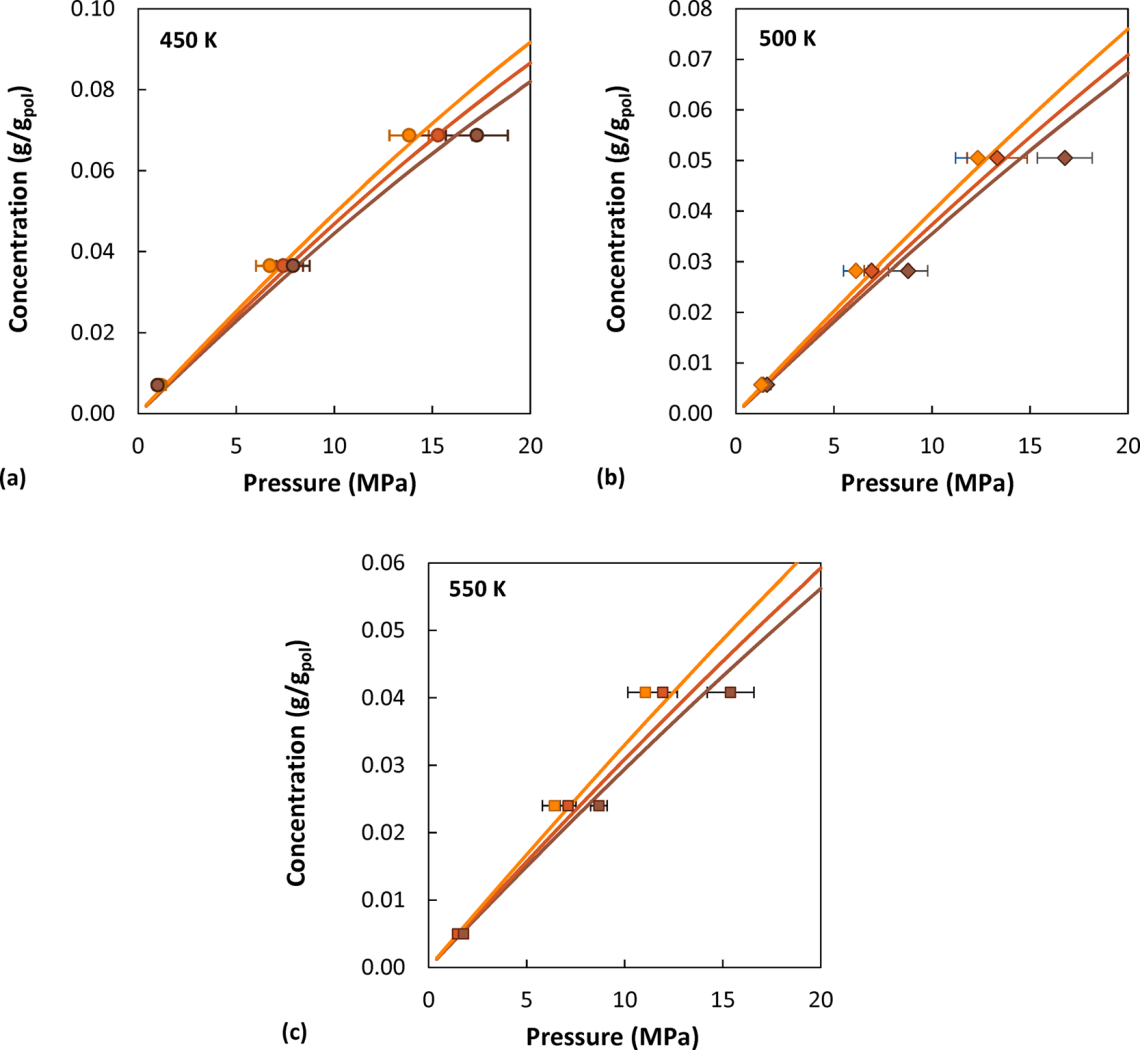
Comparison between simulated sorption isotherms and the results obtained with the SL EoS (solid lines). Circles represent simulated data at 450 K, diamonds at 500 K, and squares at 550 K. A molecular weight of 2100 g/mol is depicted in orange, 5200 g/mol in red, and 31000 g/mol in brown. For data points at low concentrations the error bar is smaller than the symbol size.

In Figure 15, the simulation results are compared with the predictions of the Sanchez–Lacombe EoS. The EoS results would suggest a weaker *M*_w_ dependence at high pressure, especially at 500 and 550 K, compared to the simulated results. At higher pressure the difference between the density predicted by the simulations and calculated with the equation of state is slightly higher (average deviation ∼3%) compared to the pure polymer cases (average deviation ∼2%). However, the high-*M*_w_ system generally displays lower solubility compared to the EoS results, consistent with the fact that the simulations predict higher density of the system. However, the low- and intermediate-*M*_w_ systems display comparable or even higher solubility, despite the fact that they are also denser.

In the Supporting Information results obtained for the Henry’s law constant, *H*, are also reported (Figure S19). They were calculated from Widom’s test particle insertions performed on the pure polymer systems. The calculated values for the excess chemical potential were used to evaluate the Henry’s law constant by means of eq 3. The simulated values are higher than the experimental results by Sato and co-workers^23^ by approximately a factor of 2. The discrepancy is a result of an underestimation of the excess chemical potential. At 500 K, a value of μ_CO_2__^ex^ = 3.8 kJ/mol was obtained for the low-*M*_w_ system, 3.4 kJ/mol for the intermediate-*M*_w_ system, and 3.1 kJ/mol for the high-*M*_w_ system. These values are about 3–4 times lower than the value of 11.77 kJ/mol reported by Eslami et al.^44^ for simulated CO_2_ sorption in polystyrene at the same temperature. In the infinite dilution regime at 450 K their values for Henry’s law constant are 3 times higher than the corresponding experimental values, while their calculated sorption isotherms at 298 and 373 K are in good agreement with measurements.

From the slope of an Arrhenius plot of ln(1/*H*) vs 1/*T* it is possible to evaluate the enthalpy of sorption. The result obtained for the experimental data of Sato et al.^23^ is 9.6 kJ/mol. From our simulations a value of 13.9 kJ/mol was obtained in the case of the high-*M*_w_ system. Simulated data at 2100 and 5200 g/mol are more scattered, but values of 13.0 and 12.8 kJ/mol were obtained by linear interpolation for the enthalpy of sorption of the low and intermediate *M*_w_, respectively. The trends are displayed in Figure S20. With the SL EoS, a value of 8.6 kJ/mol is obtained for the enthalpy of sorption at all molecular weights, which is in good agreement with the experimental value. Even though both the simulations and the SL EoS overestimate the value of the solubility coefficients in the zero-pressure limit, the EoS captures the temperature dependence more accurately. Nonetheless, it must be noticed that simulations present several advantages with respect to an equation of state. First, since in several cases the force field parameters can be obtained also from quantum-mechanical calculations and not through the use of empirical adjustable parameters, they can have a higher predictive power. Second, when general-purpose force fields are used, the parameters refer to atomic species belonging to a functional group and are transferable between different molecules, whereas in the case of an equation of state, each molecule has its own parameter set, which is not transferable. Finally, the strength of a molecular approach is that the same model and molecular representation allow to predict not only macroscopic thermodynamic quantities like density, swelling, sorption, as in the case of the EoS, but also dynamical quantities, like the diffusivity reported hereafter, and structural features like the structure factor and RDFs. Therefore, while an equation of state can provide a faithful representation of the physical properties of materials, it cannot capture the physical mechanisms that govern their macroscopic behavior. Thus, molecular simulations provide more insight into the actual processes than mere description of experimental observations.

### Diffusivity

From the mean-squared displacement (MSD = (*R*_*i*_(*t*) – *R*_*i*_(0))^2^) of CO_2_ molecules along the *NVE* trajectories, the CO_2_ self-diffusion coefficient was evaluated in the Fickian regime through the Einstein relation:
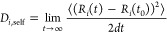
8In the previous relation, *R*_*i*_(*t*) is the position of the center of mass of a CO_2_ molecule at time *t*, *R*_*i*_(*t*_0_) is its position at an initial time (multiple time origins were considered in the average), and *d* is the dimensionality of the system, 3 in the present case. The logarithm of MSD was plotted against the logarithm of time to identify the Einstein diffusion regime region, characterized by a slope equal to 1 of log(MSD) vs log(*t*). Self-diffusivities are a good approximation of binary diffusivities in the case of an infinitely dilute system. Because in this study higher concentration values were considered, binary diffusion coefficients were calculated from the values of the self-diffusivities of CO_2_:^90^
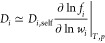
9*w*_*i*_ represents the gas mass fraction and *f*_*i*_ its fugacity. In the previous relation it is assumed that the polymer diffusivity is negligible in comparison to that of the gas. At the lower and intermediate temperatures, the Fickian diffusion regime was not reached by the polymer during the simulation; therefore, the calculation of polymer diffusivities is not warranted. On the other hand, such an evaluation was possible at the highest temperature, and it was indeed verified that the polymer diffusivities were 3 and 4 orders of magnitude lower than those of the gas for the cases of *M*_w_ 2100 and 5200 g/mol. In the high-*M*_w_ case, the polymer did not reach a Fickian diffusion regime in the time of the simulation, even at 550 K; therefore, the diffusivity could not be extracted. Because the sorption isotherms have a linear shape, the correction introduced by the thermodynamic factor in eq 9 is small: it is lower than 10% in all cases, except at 450 K, where it is 18% for the 5200 g/mol case and 24% in the 31200 g/mol case. Self-diffusion coefficients obtained for CO_2_ and aPS are reported in Table S10.

**Figure 16:**
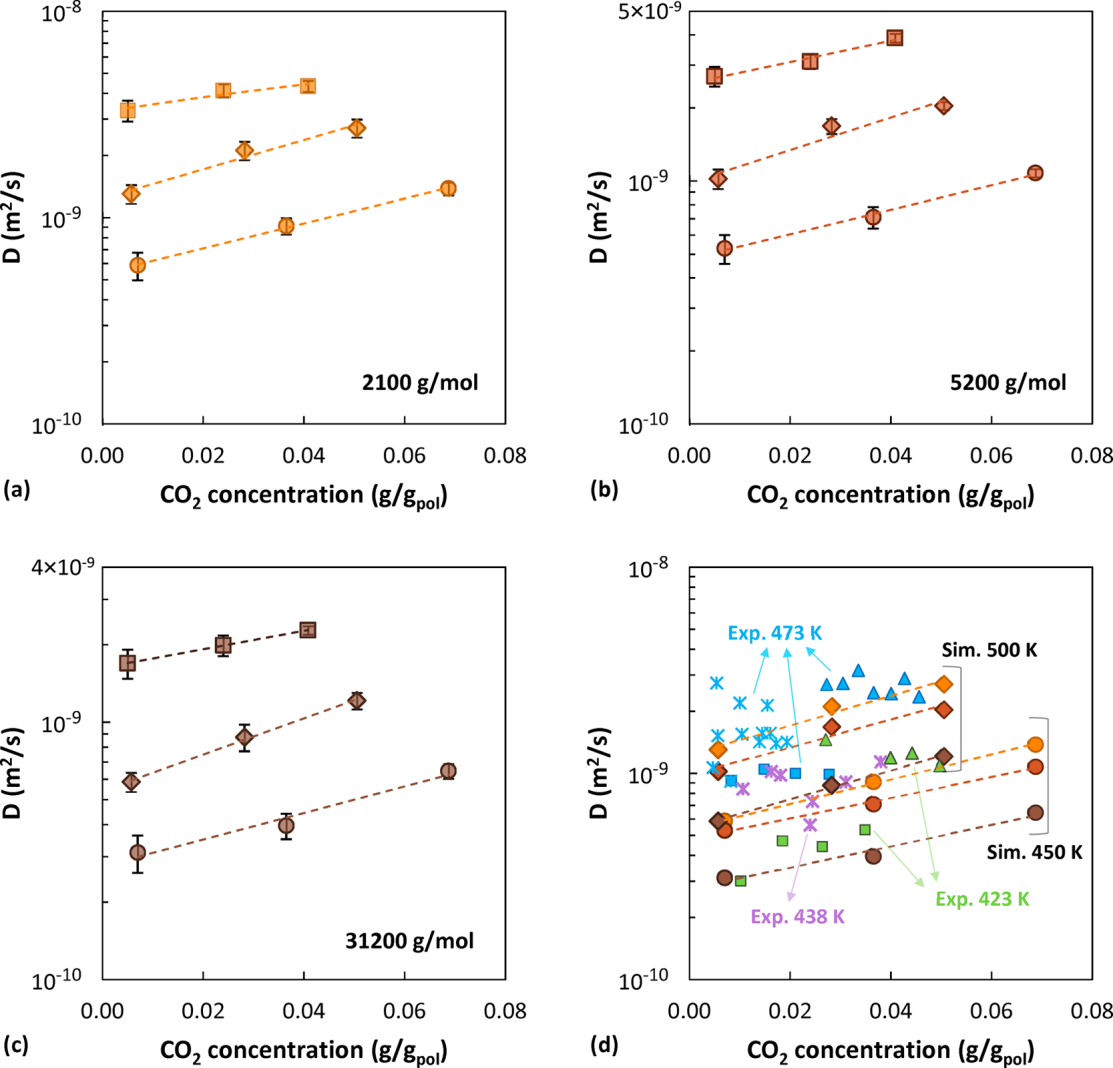
CO_2_ diffusion coefficients in atactic polystyrene as a function of concentration, at different temperatures and *M*_w_. Circles: 450 K. Diamonds: 500 K. Squares 550 K. Orange: *M*_w_ of 2100 g/mol. Red: 5200 g/mol. Brown: 31000 g/mol. (d) Comparison with experimental CO_2_ diffusion coefficients. Squares are data from ref (23) (*M*_w_ of 330000 g/mol), triangles from ref (20) (*M*_w_ of 250000 g/mol), and stars from ref (24) (*M*_w_ of 168000 g/mol). Blue represents data at 473 K, green at 423 K, and purple at 438 K. Error bars were calculated with the block average method and in some of the cases are of the same size of the symbols.

Figure 16 shows the calculated binary diffusion coefficients as a function of gas concentration at different temperatures and molecular weights. An exponential growth of diffusivity with respect to concentration is found at all conditions with comparable slopes in the systems of different *M*_w_ at the same temperature. Diffusivity consistently increases with increasing temperature and decreasing *M*_w_. Figure 16 shows also the comparison with experimental data. The three data sets used for comparison are for high-*M*_w_ aPS samples at two different temperatures: 423 and 473 K from the work of Areerat et al.^20^ and 438 and 473 K from the work of Perez-Blanco et al.^24^ Therefore, there is approximately the same temperature difference between the two series and the simulated data presented for the case 450 and 500 K for all *M*_w_. Indeed, the difference between data at two temperatures is consistent between simulations and experiments. Simulation results are in the same order of magnitude as the experimental data, located in between the different sets. The comparison is also satisfactory with older data measured by Newitt et al.:^18^ at 440 K and 0.005 g_CO_2__/g_pol_, *D*_CO_2__ is 3.9 × 10^–10^ m^2^/s; at 450 K and 0.005 g_CO_2__/g_pol_, *D*_CO_2__ is 2.45 × 10^–10^ m^2^/s; and at 460 K and 0.005 g_CO_2__/g_pol_, *D*_CO_2__ is 2.11 × 10^–10^ m^2^/s. Taking into account also the scattering of the experimental data at the same temperature, the agreement with the simulated results is good.

## Conclusions

In this study, molecular simulations were applied to the study of a polymeric system containing a plasticizing agent in full atomistic detail, and an extensive mechanistic analysis of the underlying microscopic phenomena was conducted. Atactic polystyrene chains were generated and equilibrated up to high molecular weights, through a multiscale equilibration procedure for the case of the systems at the highest molecular weight. The pure polymer and gas–polymer systems thus obtained were simulated and analyzed to calculate a wide range of properties, at both the macroscopic and the microscopic level: density, radius of gyration, radial distribution functions, X-ray scattering patterns, CO_2_ sorption, CO_2_-induced swelling, CO_2_ diffusion coefficients, and local dynamics of the polymer. The effect of temperature, molecular weight, and gas concentration on the aforementioned properties was systematically assessed and presented. The calculated quantities were compared to experimental data, when available, or to the predictions of the Sanchez–Lacombe EoS, which was specifically reparametrized to capture the molecular weight dependence of the macroscopic properties more accurately. The density of the system was slightly overestimated in the simulations at all *M*_w_, while the temperature dependence and chain dimensions were in good agreement with experimental measurements. The local structure characteristics of the simulated systems were found to be in very close agreement with the experimental results, and the contributions of different segments of the chain to the structural features provided a detailed interpretation of their origin. In the case of gas−polymer systems, it was found that CO_2_ affects interchain packing more significantly than the average chain dimensions.

The interaction potential resulted in slower segmental dynamics, compared to experiments, but consistent and meaningful trends with respect to the variables considered were calculated. The local dynamics of the matrix is faster at higher gas concentration, which is a manifestation of the plasticization effect induced by the presence of CO_2_; the more mobile system at lower *M*_w_ is affected to a greater extent.

The agreement between gas diffusion coefficients obtained from the mean-squared displacement of CO_2_ molecules and experimental results was good, also in terms of temperature and concentration dependence. Therefore, the ability to provide a faithful representation of the structural properties was sufficient to obtain a reliable estimate of gas diffusivity, even though characteristic times of the polymer dynamics were overestimated.

The iterative scheme adopted for the calculation of solubility allowed the prediction of sorption isotherms up to high pressures, which are difficult to reach experimentally, with rapid convergence. Moreover, it enabled the study of the penetrant induced swelling as a function of concentration.

Molecular modeling can be employed for a predictive investigation of materials properties in systems of practical technological interest. A wealth of detailed and reliable information about the microscopic characteristics and on the macroscopic behavior of a system can be extracted by the implementation of molecular simulation strategies. The application of these methods to gas–polymer properties prediction and the elucidation of the underlying molecular mechanisms is thus very appealing for the design of supercritical CO_2_ processes, efficient membrane separations, and barrier materials for packaging.

## Supporting Information Available

Potential energy parameters for atactic polystyrene (aPS) and CO_2_; description of the Sanchez–Lacombe equation of state (EoS) parameters regression procedure; additional results on volumetric properties, radial distribution functions, segmental dynamics, solubility, and diffusivity (PDF)
