# Supplementary material for: Molecular Simulations and Mechanistic Analysis of the Effect of CO2 Sorption on Thermodynamics, Structure, and Local Dynamics of Molten Atactic Polystyrene
Source: Macromolecules. 2020 May 11;53(10):3669–89. doi: 10.1021/acs.macromol.0c00323 (PMC8016389; doi:10.1021/acs.macromol.0c00323)
Supplement: Supplementary file 1 — ma0c00323_si_001.pdf [file ma0c00323_si_001.pdf]

Molecular Simulations and Mechanistic Analysis of the Effect of CO_2 Sorption on Thermodynamics, Structure, and Local Dynamics of Molten Atactic Polystyrene

Eleonora Ricci[1], Niki Vergadou[2*], Georgios G. Vogiatzis[3], Maria Grazia De Angelis[1], Doros N. Theodorou[4]*

[1] Department of Civil, Chemical, Environmental and Materials Engineering, University of Bologna, Italy

[2] Institute of Nanoscience and Nanotechnology, National Center for Scientific Research "Demokritos", Athens, Greece

[3] Department of Mechanical Engineering, Eindhoven University of Technology, The Netherlands

[4] School of Chemical Engineering, National Technical University of Athens, Greece

- Supporting Information -

Contents

Potential Energy Parameters for Atactic Polystyrene (aPS) and CO_2 3
Sanchez-Lacombe Equation of State (EoS) Parameters Regression 6
Density 12
Thermal expansion coefficients 13
Isothermal compressibility 14
Radius of Gyration 15
Radial Distribution Functions between Polystyrene atoms 17
Radial Distribution Functions between CO_2 and Polystyrene atoms 21
Segmental Dynamics 23
Dynamics of Different Chain Segments 27
Dynamics of End-to-end Vectors 30
Henry's Law Constant 31
Enthalpy of Sorption 33
Self-diffusion coefficients 34
References 35

Potential Energy Parameters for Atactic Polystyrene (aPS) and CO_2

An all-atom representation was adopted, using the potential energy parameters from the work of Müller-Plathe[S1] for aPS, in conjunction with harmonic constants for bond stretching from the work of Ndoro et al.,[S2] while the EPM2 model was chosen for CO_2.[S3] Instead of a rigid representation, the fluctuations of the bond length of CO_2 were kept as low as possible by using a harmonic potential with a high value for the constant k_b. It was verified that in this way the bond length of CO_2 deviated by less than 0.01% from its equilibrium value of 1.1490 Å.

Table S1. EPM2 force field parameters for CO_2.[S3]

Nonbonded Interactions	$U_{NB}(r_{ij}) = 4\varepsilon\left[\left(\frac{\sigma}{r_{ij}}\right)^{12} - \left(\frac{\sigma}{r_{ij}}\right)^{6}\right] + \frac{q_i q_j}{4\pi\epsilon_0 r_{ij}}$		
	ε (kcal/mol)	σ (Å)	q (e)
C_{CO2}	0.0559	2.757	+ 0.6512
O_{CO2}	0.1600	3.033	–0.3256
Bond Stretching	$U_l(r_{ij}) = K_l(r_{ij} - r_0)^2$		
	$K_l\left(\frac{\text{kcal}}{\text{mol}}\right)$		r_0 (Å)
C_{CO2} – O_{CO2}	1000		1.149
Angle Vibration	$U(\phi) = K_\phi(1 - \cos\phi)$		
		$K_\phi\left(\frac{\text{kcal}}{\text{mol rad}^2}\right)$	
O_{CO2} – C_{CO2} – O_{CO2}		147.706	

Table S2. Force field parameters for non-bonded interactions of all-atom polystyrene.[S1]

Nonbonded Interactions	$U_{NB}(r_{ij}) = 4\varepsilon\left[\left(\frac{\sigma}{r_{ij}}\right)^{12} - \left(\frac{\sigma}{r_{ij}}\right)^{6}\right] + \frac{q_i q_j}{4\pi\epsilon_0 r_{ij}}$		
	ε (kcal/mol)	σ (Å)	q_i (e)
C_{AL}	0.0841	3.207	0
H_{AL}	0.0760	2.318	0
C_{AR}	0.0703	3.55	– 0.115
H_{AR}	0.0301	2.42	+ 0.115

Table S3. Force field parameters for bond stretching[S2], angle vibrations, dihedral and improper angles[S1] of all-atom polystyrene.

Bond Stretching	$U_l(r_{ij}) = K_l(r_{ij} - r_0)^2$	
	$K_l\left(\frac{\text{kcal}}{\text{mol}}\right)$	r_0 (Å)
C_{AL} – C_{AL}	310.4	1.53
C_{AR} – C_{AR}	469.7	1.39
C_{AL} – C_{AR}	317.5	1.51
C_{AL} – H_{AL}	239.0	1.09
C_{AR} – H_{AR}	239.0	1.08
**Angle Vibration**	$U(\phi_i) = K_\phi(\phi_i - \phi_0)^2$	
	$K_\phi\left(\frac{\text{kcal}}{\text{mol rad}^2}\right)$	ϕ_0 (deg)
H_{AL} – C_{AL} – H_{AL}	36.616	109.45
C_{AL} – C_{AL} – H_{AL}	43.846	109.45
C_{AL} – C_{AL} – C_{AL}	57.636	109.45
C_{AR} – C_{AL} – H_{AL}	43.846	109.45
C_{AL} – C_{AL} – C_{AR}	57.636	109.45

$C_{AL} - C_{AR} - C_{AR}$	45.005	120
$C_{AR} - C_{AR} - C_{AR}$	45.005	120
$C_{AR} - C_{AR} - H_{AR}$	50.048	120
Backbone dihedral	$U(\varphi_i) = K_\varphi(1 + \cos{(3\varphi_i)^2})$	
	$K_\varphi \left(\frac{\text{kcal}}{\text{mol}}\right)$	
$C_{AL} - C_{AL} - C_{AL} - C_{AL}$	1.434	
$C_{AL} - C_{AL} - C_{AL} - H_{AL}$ (terminal methyl only)	1.434	
Ring Dihedral	$U(\vartheta_i) = K_\vartheta(\vartheta_i - \vartheta_0)^2$	
	$K_\vartheta \left(\frac{\text{kcal}}{\text{mol rad}^2}\right)$	$\vartheta_0\ (deg)$
$C_{AR} - C_{AR} - C_{AR} - C_{AR}$	20.005	0
Impropers	$U(\theta_i) = K_\theta(\theta_i - \theta_0)^2$	
	$K_\theta \left(\frac{\text{kcal}}{\text{mol rad}^2}\right)$	θ_0 (deg)
$C^{II}_{AR} - C^{III}_{AR} - C^{I}_{AR} - H^{(on\ II)}_{AR}$	20.005	0
$C^{II}_{AR} - C^{III}_{AR} - C^{I}_{AR} - C^{(on\ II)}_{AL}$	20.005	0

Sanchez-Lacombe Equation of State (EoS) Parameters Regression

In the lattice fluid representation used to derive the Sanchez-Lacombe EoS[S4–S6], each substance is univocally characterized by three macroscopic parameters T^*, p^*, ρ^*. The **Sanchez-Lacombe EoS** (eq (S4)) is written in terms of reduced variables $\tilde{T}, \tilde{p}, \tilde{\rho}$, defined as follows ($T$, p, ρ represent the temperature, pressure and density of the system):

$$\tilde{T} = \frac{T}{T^*} \tag{S1}$$

$$\tilde{p} = \frac{p}{p^*} \tag{S2}$$

$$\tilde{\rho} = \frac{\rho}{\rho^*} \tag{S3}$$

The EoS is formally identical for pure components and mixtures, provided that the corresponding definition of the reduced variables (*i.e.* that for a pure component or that for the multicomponent case) is used:

$$\tilde{\rho} = 1 - \exp\left[-\frac{\tilde{\rho}^2}{\tilde{T}} - \frac{\tilde{p}}{\tilde{T}} - \tilde{\rho}\left(1 - \sum_i^N \frac{\phi_i}{r_i}\right)\right] \tag{S4}$$

N is the total number of components in the system. Mixing rules to obtain the macroscopic parameters and reduced variables of a mixture are the following:

$$\frac{1}{\rho^*} = \sum_i^N \frac{\omega_i}{\rho_i^*} \tag{S5}$$

$$p^* = \sum_i^N \phi_i p_i^* - \sum_i^{N-1} \sum_{j>i}^N \phi_i \phi_j \Delta p_{ij}^* \tag{S6}$$

$$T^* = \frac{p^*}{\sum_i^N \frac{p_i^* \phi_i}{T_i^*}} \quad \text{(S7)}$$

Δp_{ij}^* expresses the characteristic binary interactions between species i and j and contains an **adjustable binary paramete**r k_{ij}, to account for deviations from the geometric mean mixing rule:

$$\Delta p_{ij}^* = p_i^* + p_j^* - 2(1 - k_{ij})\sqrt{p_i^* \cdot p_j^*} \quad \text{(S8)}$$

ϕ_i represents the volume fraction of component i in closed packed conditions, and can be calculated from the knowledge of the mass fractions ω_i:

$$\phi_i = \frac{\omega_i/\rho_i^*}{\sum_i^N \omega_i/\rho_i^*} \quad \text{(S9)}$$

r_i is the number of lattice cells occupied by a molecule in a mixture. It is related to the corresponding value for the pure component, r_i^0, and the close-packed molar volume of a lattice cell for pure i, v_i^*, or for a mixture, v^*:

$$r_i = \frac{r_i^0 v_i^*}{v^*} \quad \text{(S10)}$$

$$r_i^0 = \frac{M}{\rho_i^* v_i^*} \quad \text{(S11)}$$

$$v^* = \frac{T^* R}{p^*} \quad \text{(S12)}$$

The expression for the chemical potential to be used in phase equilibria calculations is given below:

$$\frac{\mu_i}{RT} = ln(\tilde{\rho}\phi_i) - ln(1-\tilde{\rho})\left[r_i^0 + \frac{r_i - r_i^0}{\tilde{\rho}}\right] - r_i$$

$$- \tilde{\rho}\frac{r_i^0 v_i^*}{RT}\left[p_i^* + \sum_{j=1}^{N} \phi_i\left(p_j^* - \Delta p_{i,j}^*\right)\right] + 1 \qquad \text{(S13)}$$

The pressure-volume-temperature data reported by Zoller and Walsh[S7] for polystyrene was used as target to obtain a functional dependence of the SL EoS parameters on M_w. The comparison between experimental data and EoS results is shown in **Figure S1** and the parameters obtained for each case are reported in **Table S4**. A good fit could be obtained at all conditions, especially at lower pressures. The maximum deviation between experimental data and EoS results was 1.1% at 110000 g/mol, 1.0% at 34500 g/mol and 9000 g/mol, and 2.5% at 910 g/mol. The average deviation was 0.4% in all cases.

Table S4. Sanchez-Lacombe EoS parameter sets for polystyrene at different M_w obtained from the best fit of data of Zoller and Walsh.[S7] 95% confidence intervals accounting for coupling between the parameters were estimated using a bootstrap method.[S8]

M_w (g/mol)	T^* (K)	P^* (MPa)	ρ^*(g/cm^3)
110000	750.0 ± 10	366 ± 26	1.096 ± 0.005
34500	749.5 ± 8	370 ± 24	1.094 ± 0.004
9000	748.5 ± 9	378 ± 27	1.088 ± 0.004
910	737.0 ± 10	400 ± 30	1.050 ± 0.006

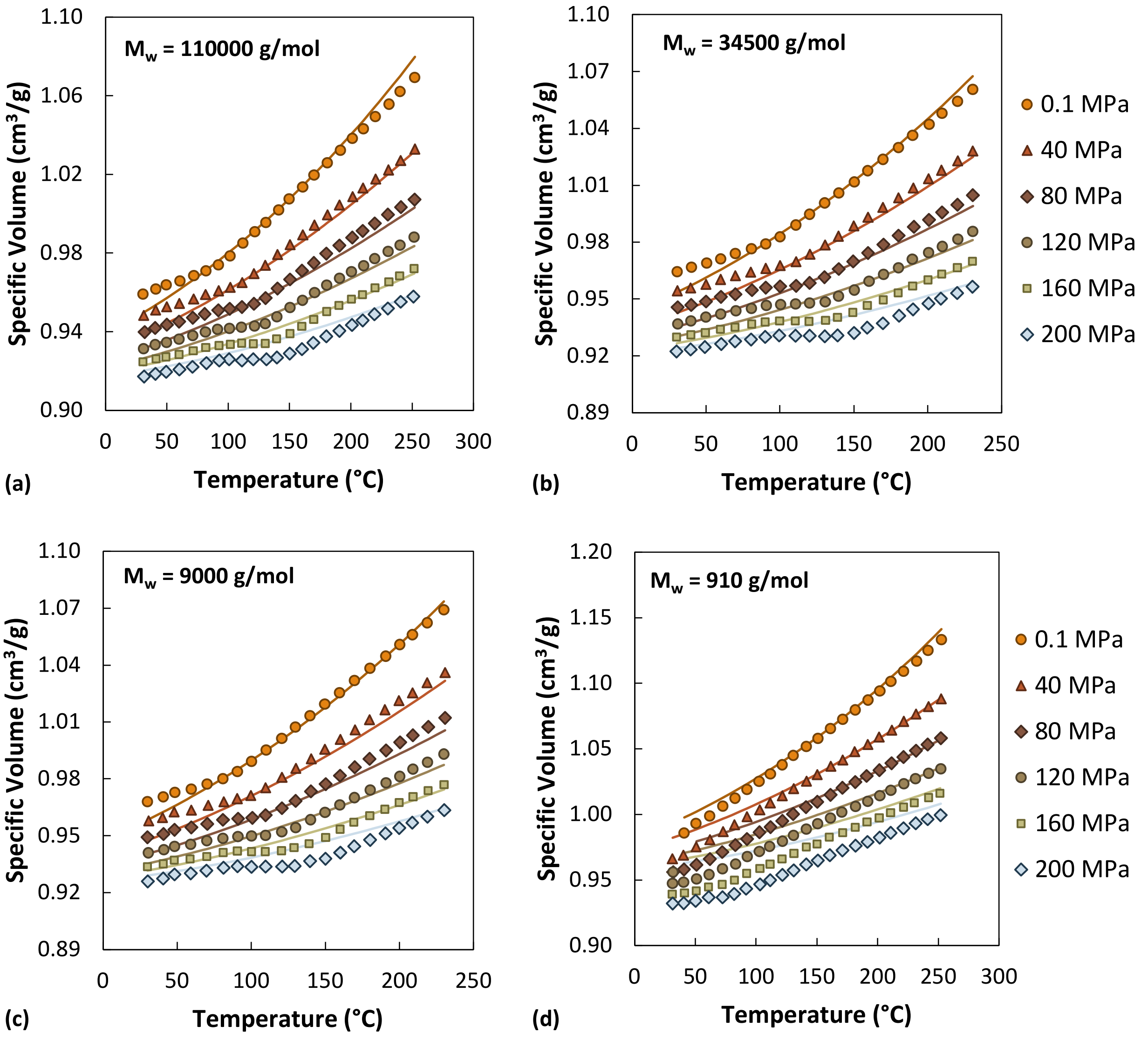

Figure S1. *pVT* curves for aPS at different M_w together with SL EoS results calculated using the parameters reported in **Table S4**.

The M_w dependence of the parameters is shown in **Figure S**. The results for T^* and ρ^* were interpolated using eq (S14), while for p^* eq (S15) was used.

$$f(x) = \frac{abx}{1 + bx} \tag{S14}$$

$$f(x) = \frac{a}{x^b} + c \tag{S15}$$

The values for the best fit constants are: a = 750 ± 10 and b = 0.063 ± 0.02 for T^*, a = 470 ± 35, b = 0.36 ± 0.04 and c = 360 ± 20 for p^*, a = 1.099 ± 0.003 and b = 0.023 ± 0.002 for ρ^*. T^* is expressed in K, p^* in MPa, ρ^* in g/cm^3 and M_w in g/mol. In the high M_w limit, these relations yield the parameter set from Doghieri and Sarti.[S9] Using these relations, the parameters corresponding to the M_w of the simulated systems were obtained and they are reported in **Table 2** in the main text.

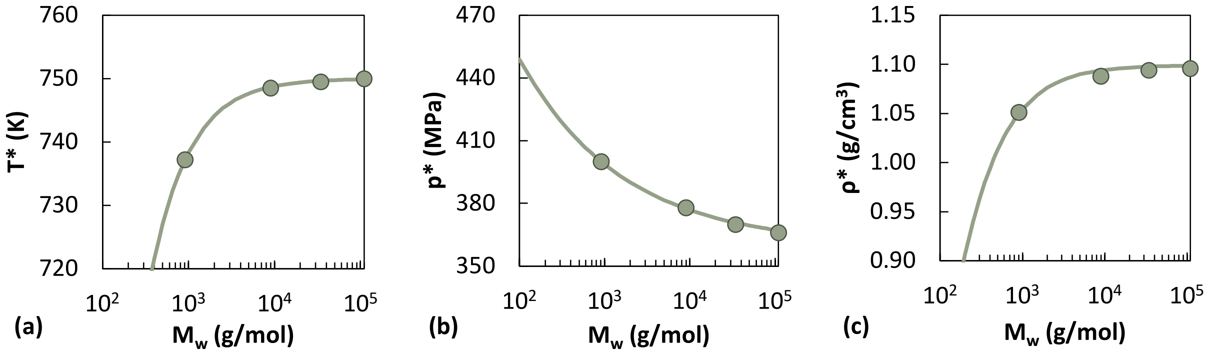

Figure S2. M_w dependence of Sanchez-Lacombe EoS parameters for aPS. The lines represent interpolations obtained with eq (S14) for (a) $T^*$and (c) ρ^* and eq (S15) for p^* (b).

Furthermore, in order to calculate the properties of aPS-CO_2 systems, the value for the binary interaction parameter k_{ij} must be determined. Since this parameter is usually temperature dependent, its value as well as its temperature dependence were determined by the best fit of the sorption isotherms reported by Sato et al.[S10] in the temperature range 373 – 453 K. This data set was selected because the sorption isotherm at 453 K showed the best agreement with the simulated data. For CO_2 the SL parameter set reported by Doghieri and Sarti[S11] was used. The binary interaction parameter is assumed to be independent of M_w and concentration. A linear extrapolation of the parameter values to the temperatures used in the simulations yielded the following values of the aPS-CO_2 binary interaction parameter: $k_{ij} = -0.041$ at 450 K, $k_{ij} = -0.060$ at 500 K, $k_{ij} = -0.080$ at 550 K. This temperature dependence, however, led to a deviation from the expected Arrhenius behavior of the solubility as a function of temperature in the high-temperature range, as can be seen in **Figure S20**. The expected trend could be obtained by extrapolating the values of k_{ij} using a quadratic temperature dependence instead of a linear one:

$$k_{ij} = 1.69 \cdot 10^{-4}\, T^2 - 1.84 \cdot 10^{-3}\, T + 4.44 \cdot 10^{-1} \tag{S16}$$

In this way, a value of $k_{ij} = -0.041$ was used at 450 K, $k_{ij} = -0.052$ at 500 K, $k_{ij} = -0.055$ at 550 K.

The EoS predictions of volumetric properties are almost insensitive to this choice, while in the case of CO_2 solubility, a maximum 15% difference is obtained at 550 K (where the difference between the two values of k_{ij} is the greatest).

Density

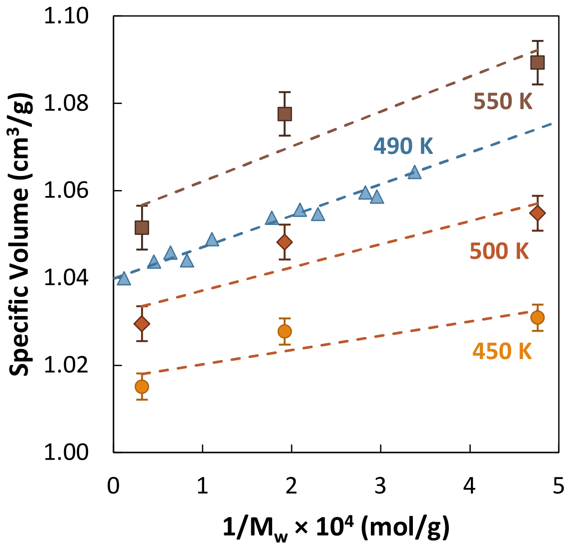

Figure S3. Specific volume of aPS as a function of inverse M_w. Orange circles are simulations at 450 K, red diamonds at 500 K, brown squares at 550 K. Results of Fox et al.[S12] at 490 K included for comparison.

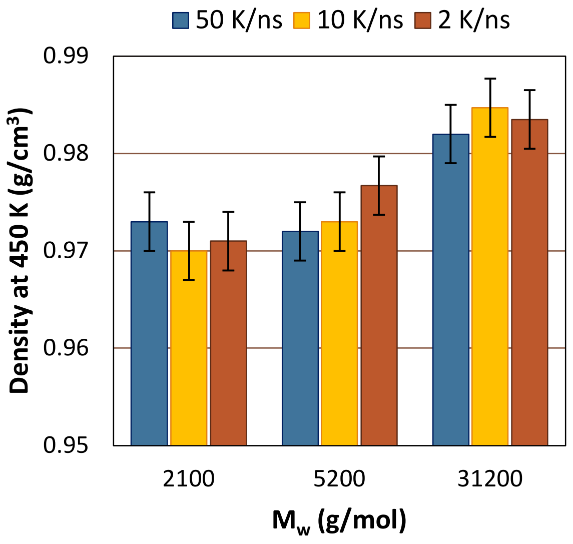

Figure S4. Effect of cooling rate on the polymer density at different M_w.

Thermal expansion coefficients

The temperature dependence of the density was assessed by calculating the **thermal expansion coefficients** at atmospheric pressure for the pure polymer systems using eq (S17). The results are reported in **Table S5** and compared to the values calculated for all the experimental and simulated data sets reported in **Figure 1** in the main text.

$$\kappa_p = -\frac{1}{V}\frac{\partial V}{\partial T}\bigg|_p \tag{S17}$$

Table S5. Thermal expansion coefficient of polystyrene at atmospheric pressure

Experimental	M_w (g/mol)	κ_p (K^{-1})
Quach, Simha[S13]	279000	5.23×10^{-4}
Ougizawa et al.[S14]	100000	5.91×10^{-4}
Zoller, Walsh[S7]	110000	5.74×10^{-4}
Zoller, Walsh[S7]	34500	5.87×10^{-4}
Zoller, Walsh[S7]	9000	5.89×10^{-4}
Zoller, Walsh[S7]	910	6.81×10^{-4}
Simulated	M_w (g/mol)	α_p (K^{-1})
Eslami, Müller-Plathe[S15]	21000	6.53×10^{-4}
Han, Boyd[S16]	8300	6.07×10^{-4}
Lyulin, Michels[S17]	8300	6.03×10^{-4}
Ndoro et al.[S18]	2100	6.14×10^{-4}
Fritz et al.[S19]	1000	4.96×10^{-4}
This work	31000	3.53×10^{-4}
	5200	4.74×10^{-4}
	2100	5.52×10^{-4}

Isothermal compressibility

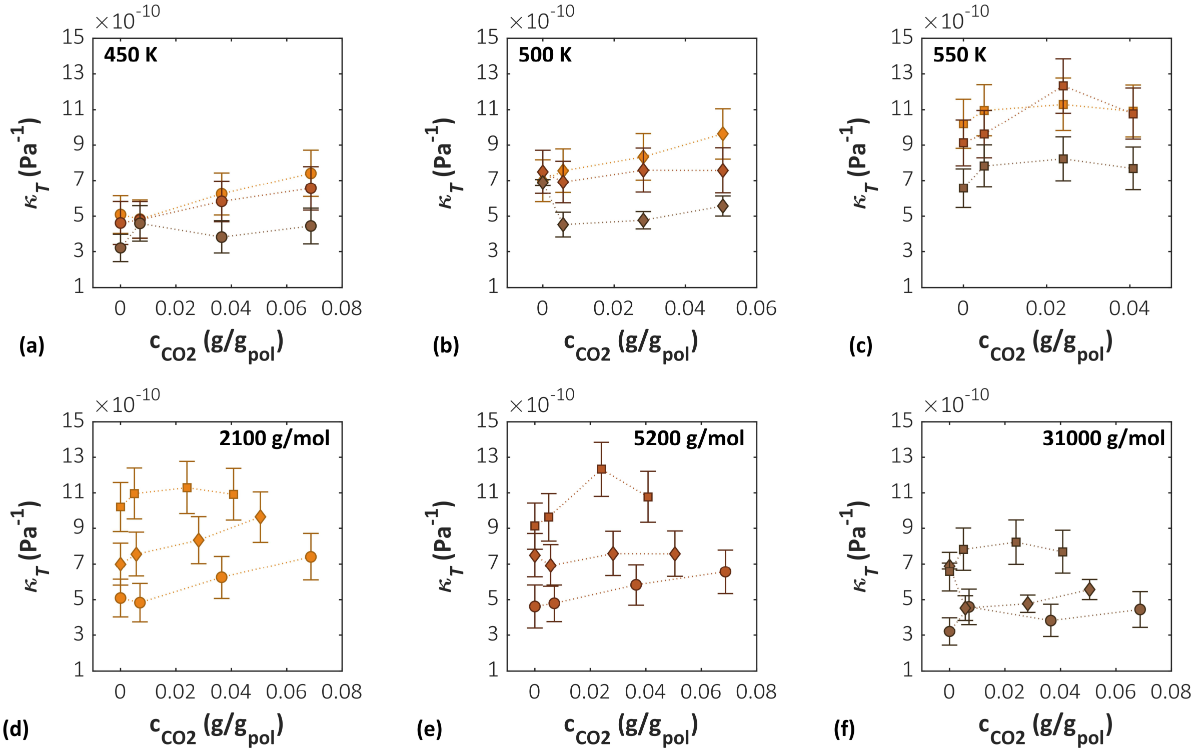

Figure S5. Isothermal compressibility as a function of CO_2 concentration. Plots (a), (b) and (c) show the effect of molecular weight at fixed temperature, plots (e), (f) and (g) show the effect of temperature at fixed molecular weight. Circles represent data at 450 K, diamonds at 500 K, squares at 550 K. Molecular weight of 2100 g/mol is depicted in orange, 5200 g/mol in red and 31000 g/mol in brown. Error bars were calculated evaluating the propagation of the statistical uncertainty associated to $\langle V^2 \rangle$, $\langle V \rangle$, and T when κ_τ is obtained using Eq. (4) in the main text. Temperature fluctuations provide a negligible contribution in the calculated uncertainty compared to volume fluctuations.

Radius of Gyration

Values for the root mean squared radius of gyration $\langle R_g^2 \rangle^{1/2}$ were obtained for all the systems using eq (S18) and are reported in **Figure S6** as a function of CO_2 content.

$$\langle R_g^2 \rangle^{1/2} = \sqrt{\frac{1}{m_{tot}} \sum_{i=1}^{N} m_i (r_i - r_{com})^2} \tag{S18}$$

In the previous relation r_{com} is the position of the center of mass of a chain, r_i the position of atom i of mass m_i along the same chain and m_{tot} is the total mass of the chain. Error bars in the plots represent the standard deviation of the mean value.

The M_w dependence of $\langle R_g^2 \rangle^{1/2}$ is shown in **Figure S7** by comparing the simulated results at 450 K with values determined by neutron scattering for monodisperse aPS ranging from 21000 to 1100000 g/mol at 393 K.[S20]

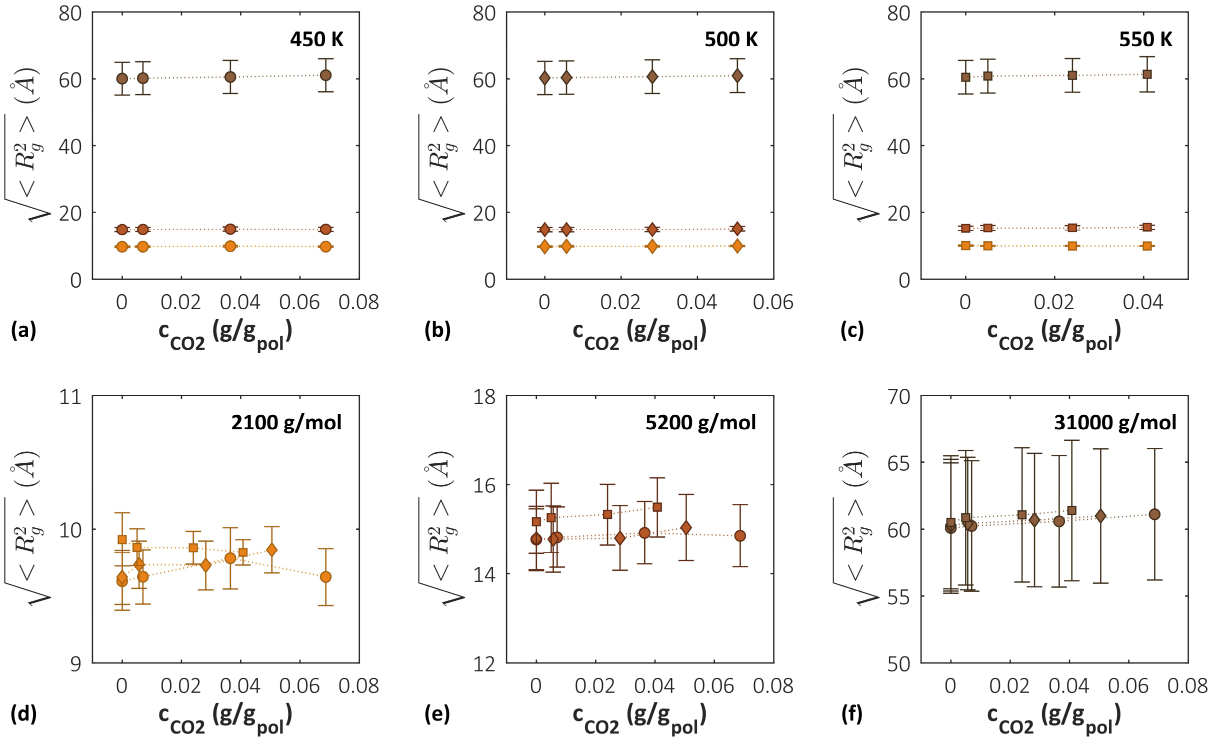

Figure S6. Radius of gyration of the polymer chains as a function of CO_2 concentration. Plots (a), (b) and (c) show the effect of molecular weight at fixed temperature, plots (e), (f) and (g) show the effect of temperature at fixed molecular weight. Circles represent data at 450 K, diamonds at 500 K, squares at 550 K. Molecular weight of 2100 g/mol is depicted in orange, 5200 g/mol in red and 31000 g/mol in brown. In subplots (a), (b), (c) error bars for the lower and intermediate M_w are of the same size of the symbols.

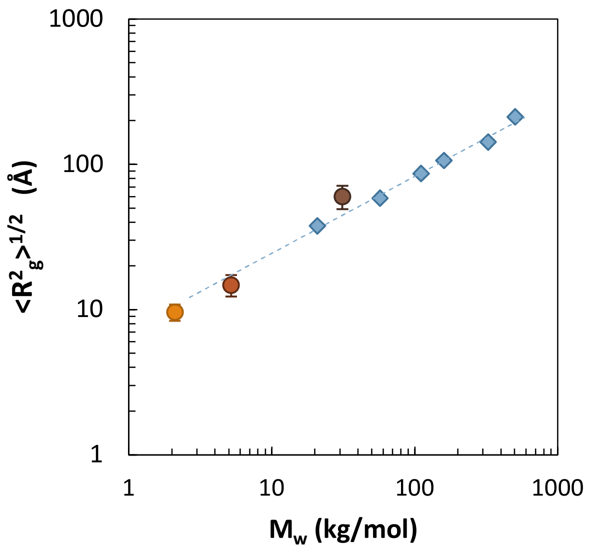

Figure S7. Comparison between the root mean squared radius of gyration of the polymer chains at 500 K and the experimental data of Cotton et al.[S20] obtained from neutron scattering experiments (blue diamonds). Circles represent the simulated values: molecular weight of 2100 g/mol is depicted in orange, 5200 g/mol in red and 31000 g/mol in brown. The error bar for the low M_w point is of the same size of the symbol.

Radial Distribution Functions between Polystyrene atoms

Experimental measurements of the RDF of carbon atoms in polystyrene showed peaks at 1.4–1.5, 2.5, 5, 6, and 10 Å.[S21,S22] The first peak is intramolecular and stems out from correlations between next neighbors, both on the chain and on the ring. These two contributions are resolved in the radial distribution functions calculated for the simulated systems (**Figure S8**): the peak at 1.39 Å represents the distance between carbon atoms on the ring, while the lower peak at ~1.5 Å includes the contributions coming from neighboring backbone carbons (bond length 1.53 Å) and from the ring carbon bonded to the backbone (bond length 1.51 Å). The second peak is also

attributed to intramolecular correlations, in this case of second neighbors in the backbone and the ring. These two contributions are fused in the simulation results; however, it is possible to attribute the shoulder on the right side of the peak to the correlations originating from the backbone, as can be seen in **Figure 5** in the main text, where the contributions from ring and backbone carbons have been calculated separately. Additionally, another peak at 2.8 Å can be recognized in the radial distribution functions of the simulated systems, which represents the correlations associated with carbons located on opposite sides of a phenyl ring. A peak is located at 3.8 Å, and it can be associated with third neighbors along the backbone. Additionally, a small feature is present at 3.2 Å, and it is associated with backbone carbon correlations.

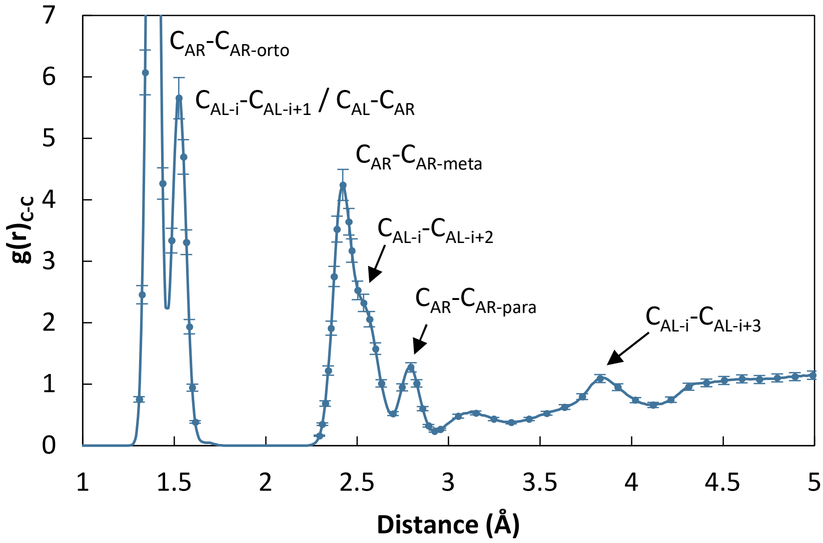

Figure S8. Radial distribution function of pairs of carbon atoms (M_w 2100 g/mol at 500 K).

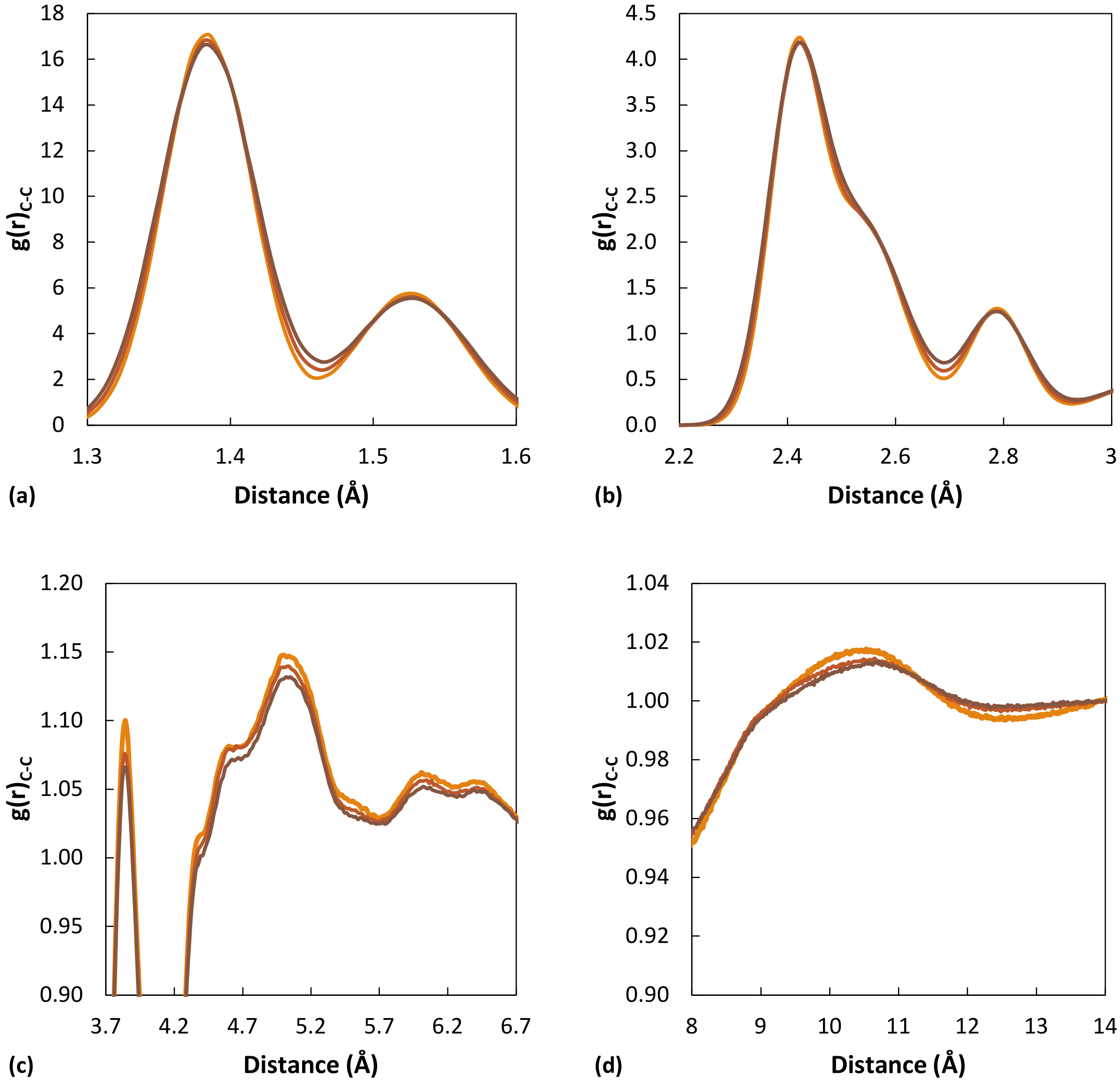

Figure S9. Effect of temperature on the peaks of the radial distribution function of pairs of carbon atoms. System: pure polymer of M_w 2100 g/mol; orange represents 450 K, red 500 K, brown, 550 K. (a) represents the short-range peaks corresponding to bonded atoms and (b) the correlations originating from second neighbors. (c) and (d) show a magnification of the features of the *g(r)* at larger distances, where also intermolecular correlations are present.

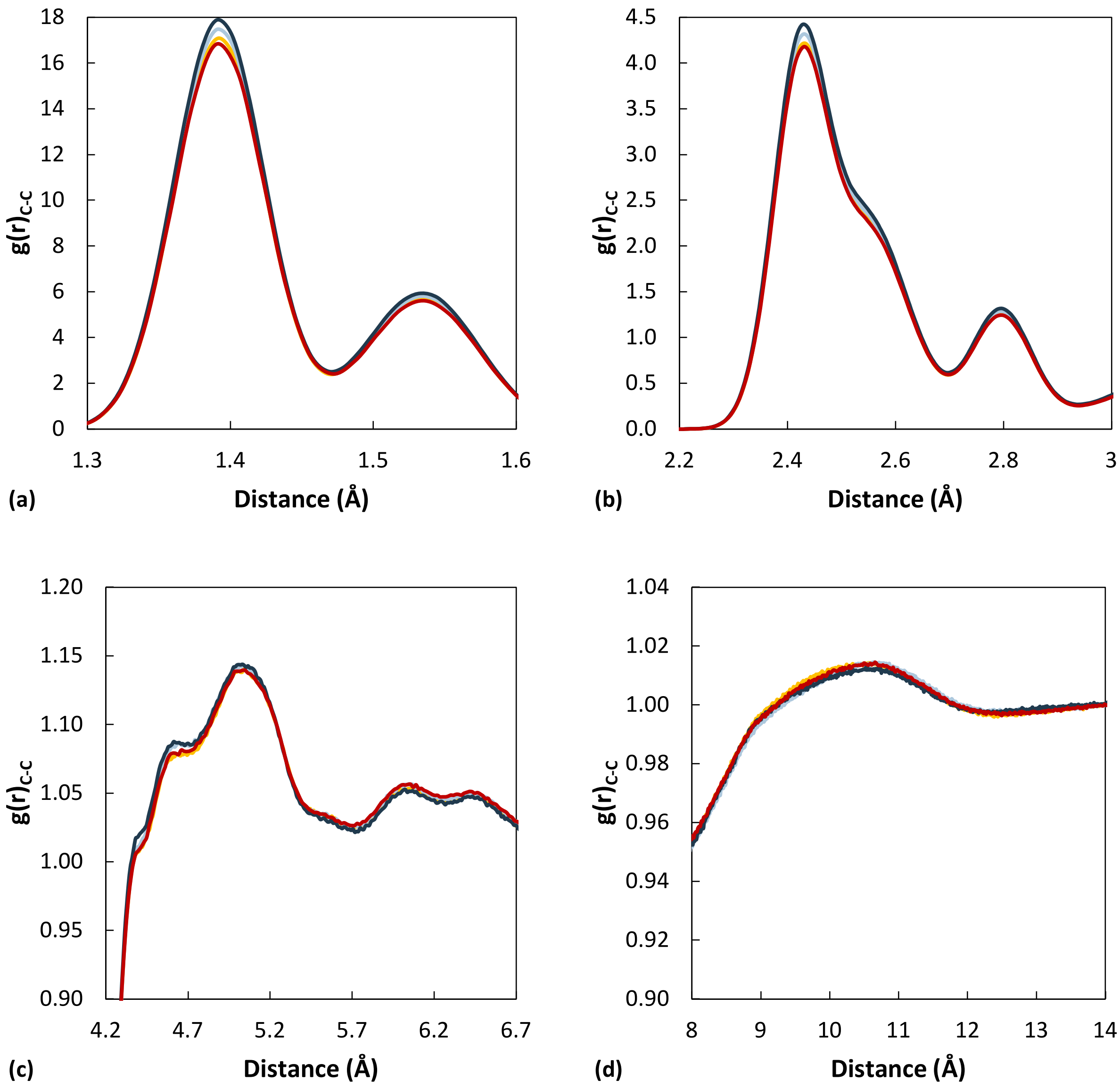

Figure S10. Effect of CO_2 concentration on the peaks of the radial distribution function of pairs of carbon atoms. System: M_w 2100 g/mol at 500K. Red represents results for the pure polymer, yellow 5.70×10^{-3} g_{CO2}/g_{pol}, light blue 2.82×10^{-2} g_{CO2}/g_{pol}, blue 5.05×10^{-2} g_{CO2}/g_{pol}. (a) represents the short-range peaks corresponding to bonded atoms and (b) the correlations originating from second neighbors. (c) and (d) show a magnification of the features of the $g(r)$ at larger distances, where also intermolecular correlations are present.

Radial Distribution Functions between CO_2 and Polystyrene atoms

Figure S11 and **Figure S12** show the effect of temperature and CO_2 concentration on the RDF of CO_2-polystyrene atom couples. With increasing temperature, the height of the peaks systematically decreases, especially in the features at lower distance, as can be observed in **Figure S11**. On the other hand, increasing CO_2 concentration does not have a discernible systematic effect (**Figure S12**). Generally, in the higher M_w systems, peaks tended to be slightly higher and broader, but this was not a systematic trend. It should be mentioned that the results at the lowest concentration suffer from worse statistics than the other cases, therefore the curves are less smooth, and it becomes difficult to establish trends in some cases.

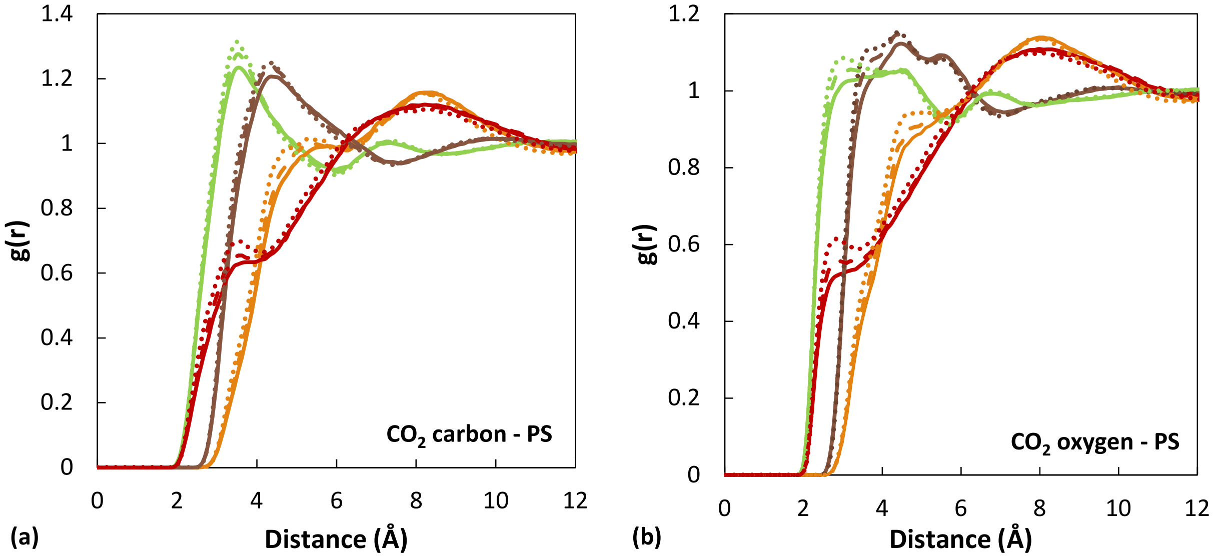

Figure S11. Radial distribution functions of pairs of CO_2-PS atoms as functions of temperature in the 5200 g/mol system at the highest CO_2 concentration studied at each temperature. The radial distribution functions of carbon atoms of the CO_2 molecule are reported in figure (a), oxygen in figure (b). Brown lines represent correlations with aromatic carbons, green with aromatic hydrogens, orange with aliphatic carbons, red with aliphatic hydrogens. Solid lines are data at 450K, dashed lines 500 K, dotted lines 550 K.

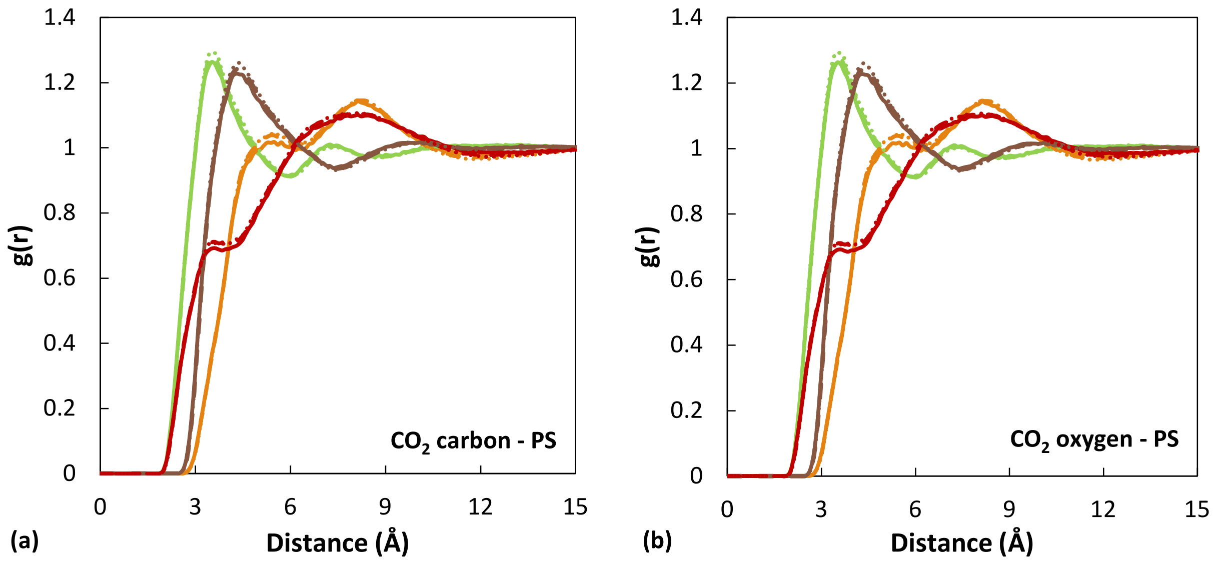

Figure S12. Radial distribution functions of pairs of CO_2-PS atoms as functions of CO_2 concentration, 2100 g/mol system at 500 K. The radial distribution functions of carbon atoms of the CO_2 molecule are reported in figure (a), oxygen in figure (b). Brown lines represent correlations with aromatic carbons, green with aromatic hydrogens, orange with aliphatic carbons, red with aliphatic hydrogens. Solid lines are data at low CO_2 concentration, dashed lines intermediate CO_2 concentration, dotted lines high CO_2 concentration.

Segmental Dynamics

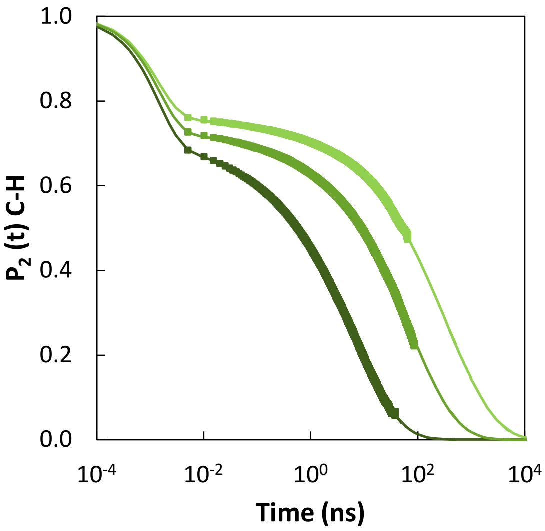

Figure S13. Effect of temperature on the orientational time autocorrelation function of the C – H bonds of pure aPS. As a representative case the 5200 g/mol aPS system is shown. Symbols represent simulation results at 450 K (light green), 500 K (green) and 550 K (dark green). Solid lines show extrapolation to shorter and longer times with a mKWW function.

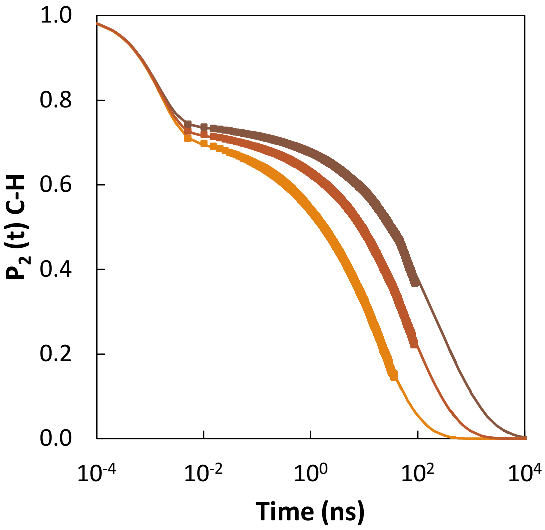

Figure S14. Effect of M_w on the orientational autocorrelation of the C – H bonds of pure aPS. The symbols represent simulated data at 500 K. Molecular weight of 2100 g/mol is depicted in orange, 5200 g/mol in red and 31000 g/mol in brown. Solid lines show extrapolation to shorter and longer times with a mKWW function.

Table S6. Best fit parameters of the mKWW equation used to represent the $P_1(t)$ of the orientational autocorrelation function of the $\mathrm{C-com_{Ring}}$ vectors.

M_w	T (K)	$c_{CO_2}(\mathrm{g/g_{pol}})$	α_{lib}	τ_{seg} (ns)	β_{KWW}	τ_{lib} (ps)	τ_c (ns)
2100 g/mol	450	0	0.04	304.92	0.65	0.40	400.99
		0.007	0.05	303.44	0.72	0.36	356.09
		0.037	0.04	114.52	0.58	0.43	172.97
		0.069	0.04	67.66	0.54	0.38	112.97
	500	0	0.05	46.32	0.58	0.44	69.34
		0.006	0.04	39.38	0.54	0.60	66.22
		0.028	0.04	14.79	0.53	0.46	25.35
		0.050	0.05	8.80	0.57	0.45	13.54
	550	0	0.04	3.65	0.57	0.43	5.70
		0.005	0.03	3.21	0.52	0.35	5.72
		0.024	0.03	2.10	0.56	0.35	3.38
		0.041	0.04	1.55	0.57	0.35	2.39
5200 g/mol	450	0	0.03	795.30	0.60	0.35	1162.19
		0.007	0.04	757.81	0.62	0.35	1052.34
		0.037	0.03	501.48	0.57	0.35	781.79
		0.069	0.04	258.64	0.53	0.35	447.54
	500	0	0.04	170.50	0.60	0.35	247.52
		0.006	0.04	115.07	0.55	0.35	189.99
		0.028	0.04	68.56	0.57	0.35	106.96
		0.050	0.04	29.02	0.59	0.35	42.81
	550	0	0.05	17.43	0.58	0.35	26.23
		0.005	0.05	14.37	0.54	0.35	24.10
		0.024	0.04	9.38	0.53	0.35	16.50
		0.041	0.04	4.64	0.56	0.35	7.38
31000 g/mol	450	0	0.03	12479.47	0.58	0.41	19092.56
		0.007	0.03	10985.94	0.59	0.36	16273.36
		0.037	0.03	6015.50	0.52	0.35	10883.53
		0.069	0.03	1886.27	0.50	0.35	3698.80
	500	0	0.04	917.56	0.51	0.35	1714.97
		0.006	0.04	1753.90	0.54	0.35	2964.46
		0.028	0.04	1066.86	0.49	0.35	2157.88
		0.050	0.04	345.97	0.49	0.35	688.75
	550	0	0.05	168.73	0.57	0.41	263.14
		0.005	0.05	147.67	0.56	0.35	232.53
		0.024	0.04	85.97	0.45	0.35	207.29
		0.041	0.04	75.43	0.48	0.35	159.67

Table S7. Best fit parameters of the mKWW equation used to represent the $P_2(t)$ of the orientational autocorrelation function of the $\mathrm{C-H}$ bonds.

M_w	$T\ (K)$	$c_{CO_2}(g/g_{pol})$	α_{lib}	τ_{seg} (ns)	β_{KWW}	τ_{lib} (ps)	τ_c (ns)
2100 g/mol	450	0	0.26	130.42	0.50	1.63	183.99
		0.007	0.25	140.15	0.55	1.48	177.51
		0.037	0.26	38.44	0.50	1.62	57.66
		0.069	0.26	23.46	0.44	1.57	44.87
	500	0	0.29	15.53	0.51	1.61	21.62
		0.006	0.28	10.11	0.48	1.50	15.59
		0.028	0.28	4.66	0.46	1.36	8.05
		0.050	0.29	2.06	0.50	1.50	2.97
	550	0	0.30	1.09	0.48	1.28	1.66
		0.005	0.29	0.95	0.46	1.17	1.56
		0.024	0.29	0.62	0.49	1.21	0.92
		0.041	0.29	0.41	0.48	1.23	0.63
5200 g/mol	450	0	0.24	337.32	0.47	1.42	580.33
		0.007	0.24	296.22	0.48	1.48	477.92
		0.037	0.25	164.95	0.47	1.49	280.02
		0.069	0.25	82.00	0.45	1.60	150.58
	500	0	0.28	57.92	0.53	1.50	76.42
		0.006	0.28	40.33	0.49	1.50	60.02
		0.028	0.28	24.08	0.50	1.58	34.60
		0.050	0.29	9.95	0.53	1.67	12.89
	550	0	0.30	5.90	0.49	1.47	8.53
		0.005	0.30	5.03	0.47	1.43	7.78
		0.024	0.29	2.90	0.46	1.34	4.87
		0.041	0.28	1.37	0.45	1.16	2.40
31000 g/mol	450	0	0.23	3664.22	0.50	1.22	5721.04
		0.007	0.23	2850.16	0.48	1.24	4664.78
		0.037	0.23	1550.49	0.44	1.38	3211.86
		0.069	0.23	882.57	0.37	1.43	2732.46
	500	0	0.25	659.23	0.46	1.50	1163.00
		0.006	0.26	569.23	0.45	1.50	1030.03
		0.028	0.26	314.26	0.42	1.39	687.03
		0.050	0.25	115.37	0.40	1.34	294.64
	550	0	0.28	66.05	0.46	1.00	111.12
		0.005	0.29	54.44	0.45	1.30	96.69
		0.024	0.26	27.23	0.36	1.07	93.13
		0.041	0.27	22.15	0.35	1.10	78.79

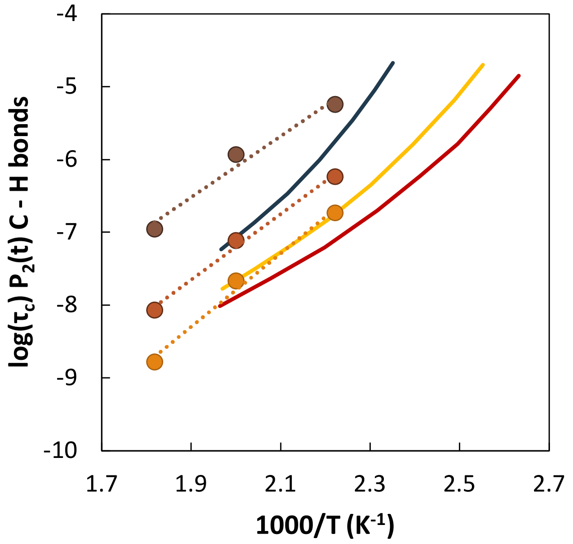

Figure S15. Segmental relaxation times for pure aPS as a function of temperature. Simulations are represented with circles: brown represents 31000 g/mol, red is 5200 g/mol, orange is 2100 g/mol. Lines are NMR measurements,[S23] arbitrarily shifted to compare the temperature dependence. Blue corresponds to a sample of 10900 g/mol, yellow 2100 g/mol, red 1600 g/mol.

Dynamics of Different Chain Segments

Table S8. Repeating units included in each subsection in the analysis of segmental dynamics as a function of the position in the chain. Subsection I is the chain end, X is the center.

M_w	I	II	III	IV	V	VI	VII	VIII	IX	X
2100 g/mol	1	2	3	4	5	6	7	8	9	10
5200 g/mol	1	2	3-4	5-7	8-10	11-13	14-16	17-19	20-22	23-25
31000 g/mol	1	2	3-10	11-30	31-50	51-70	71-90	91-110	111-130	131-150

Table S9. Relaxation times associated to the decorrelation of the $C_{ar} - H_{ar}$ bonds located at different positions in the chain at 500 K. Subsection I is the chain end, X is the center.

	**Subsection**	**I**	**II**	**III**	**IV**	**V**	**VI**	**VII**	**VIII**	**IX**	**X**
**M_w (g/mol)**	**c_{CO2} (g_{CO2}/g_{pol})**	**Relaxation time (ns)**									
2100	0	3.0	7.0	10.9	13.7	14.5	18.2	20.6	20.3	20.9	22.9
	0.006	1.5	4.1	6.9	10.3	11.9	14.0	14.7	13.9	16.1	16.2
	0.028	0.9	2.4	4.0	5.0	6.4	7.4	8.6	9.0	9.3	9.4
	0.050	0.4	1.2	2.0	2.4	2.9	3.6	3.7	3.8	3.8	4.1
5200	0	12.7	30.0	57.3	71.9	72.1	73.6	74.7	86.0	86.2	86.7
	0.006	4.8	11.2	20.3	30.6	33.2	35.1	36.8	47.1	50.6	59.2
	0.028	2.6	7.0	13.3	18.1	24.7	26.6	31.4	33.8	33.0	34.8
	0.050	1.1	3.2	8.3	14.5	14.8	14.9	13.1	15.3	14.2	15.9
31000	0	38.4	300.1	885	948	959	1068	1112	1083	1191	1230
	0.006	11.4	284.3	876.3	889.5	827.0	878.7	908.8	915.0	909.7	933.2
	0.028	9.5	183.2	513.1	646.2	598.1	630.4	668.6	661.8	618.1	678.3
	0.050	3.1	50.9	189.9	219.3	200.3	201.3	229.7	234.6	261.0	263.2

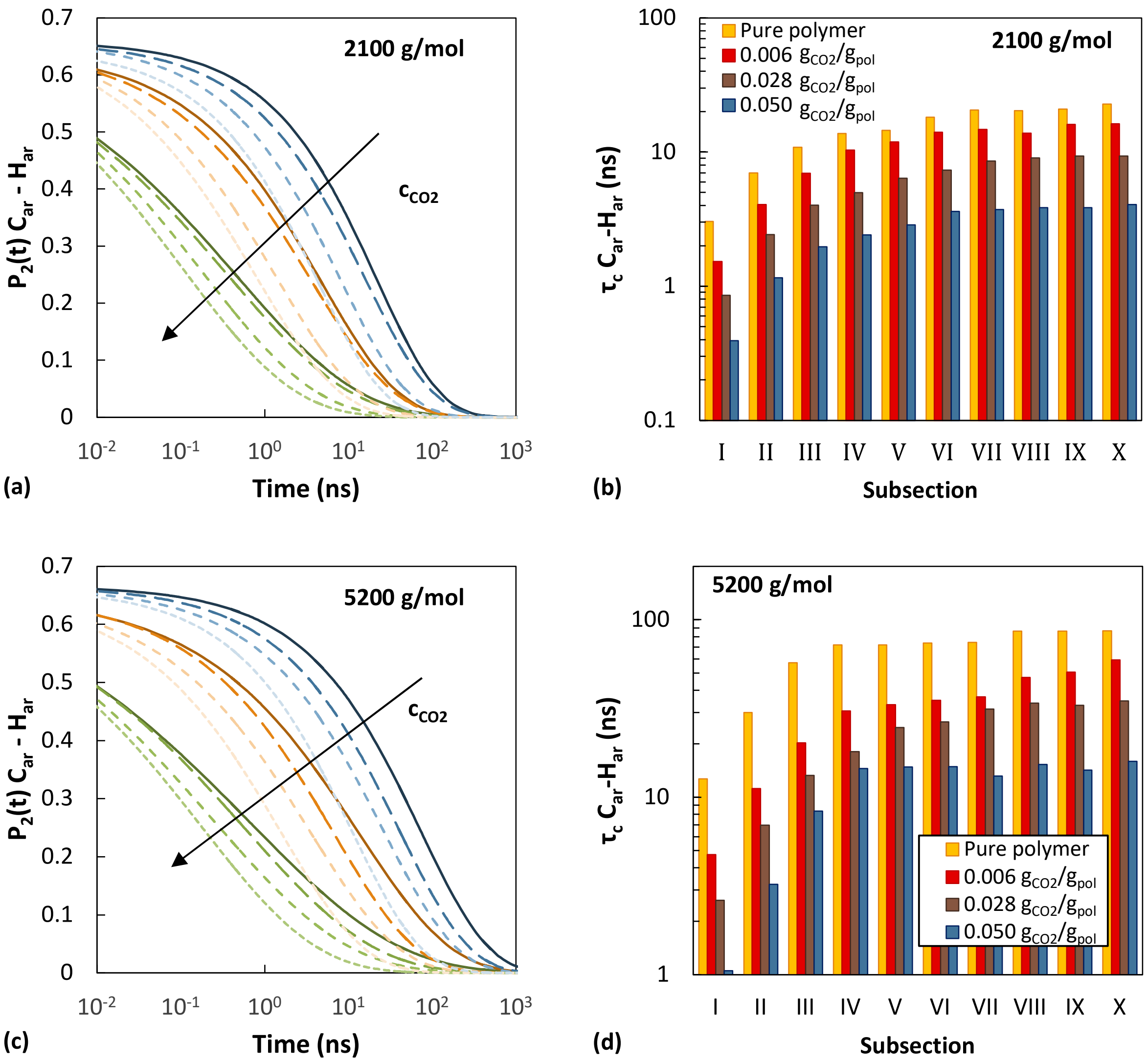

Figure S16. Effect of CO_2 concentration on the reorientational decorrelation of $C_{ar} – H_{ar}$ bonds in different chain subsections, for two polymer M_w at 500 K. **Table S8** reports how the repeating units in chains of different M_w were divided. In plots (a), (c) green represents the chain end, orange the second repeating unit starting from the chain end, and blue is the central section of the chain. Solid lines represent the pure polymer, lighter shades and shorter dash represent higher CO_2 concentration. In plots (b), (d) the relaxation times of $C_{ar} – H_{ar}$ bonds in each chain subsection are reported as functions of CO_2 concentration.

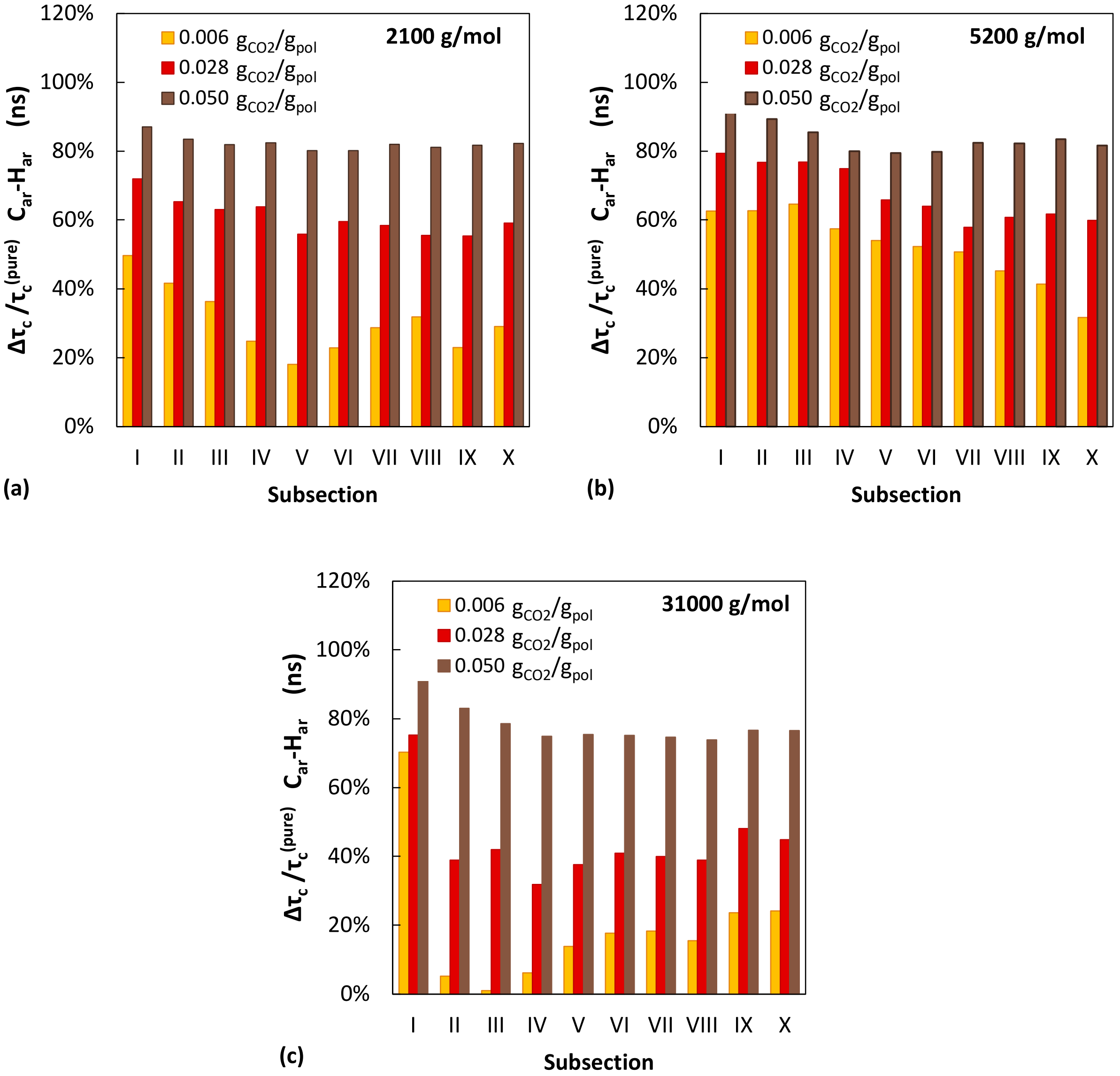

Figure S17. Relative difference in the relaxation times with respect to the pure polymer as a function of the CO_2 content in each subunit. Results for the three M_w at 500 K.

Dynamics of End-to-end Vectors

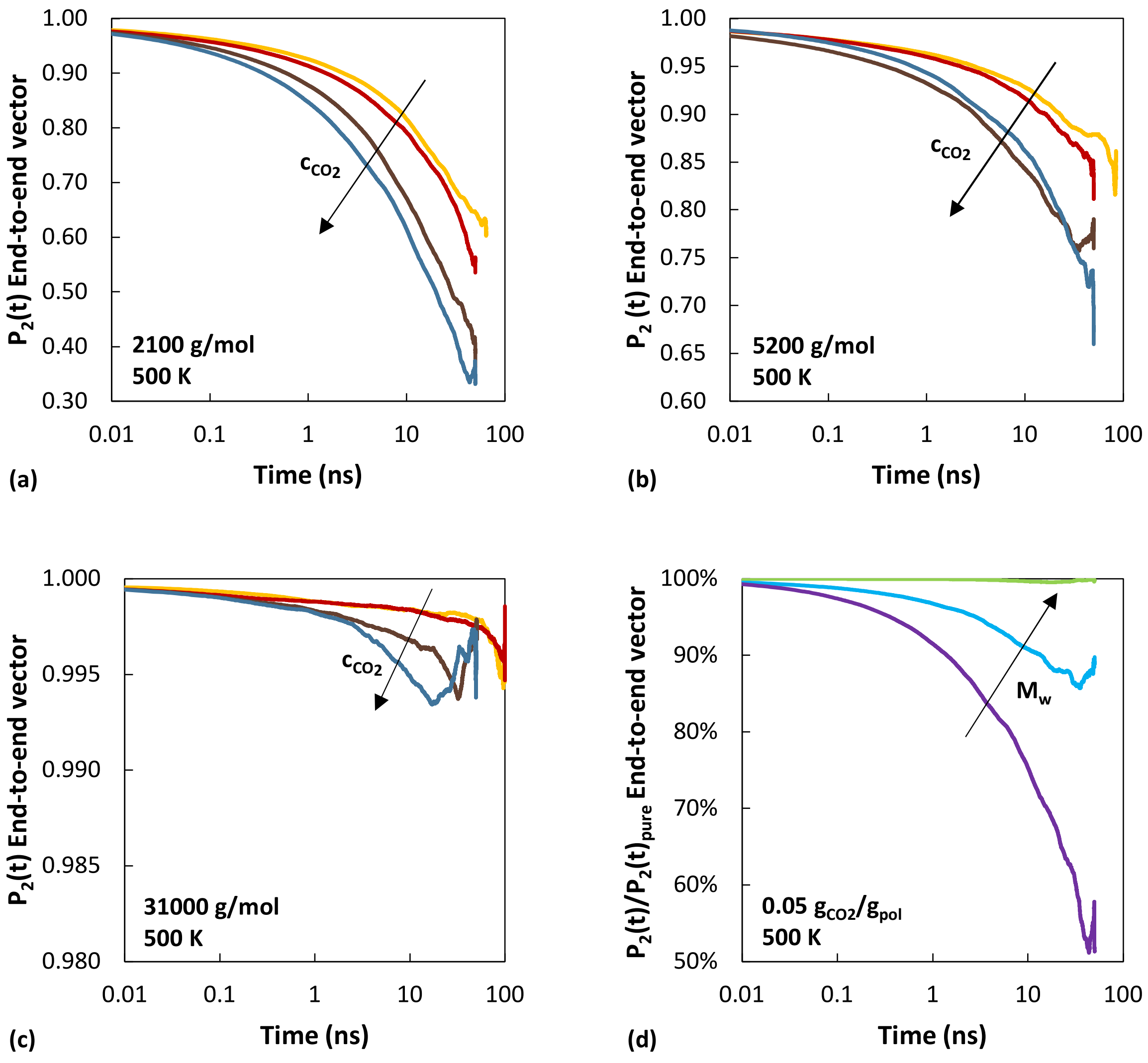

Figure S18. Effect of CO_2 concentration on end-to-end vector reorientation at 500 K and at different molecular weights. Yellow represents results for the pure polymer, red at 5.70×10^{-3} g_{CO2}/g_{pol}, brown at 2.82×10^{-2} g_{CO2}/g_{pol}, blue at 5.05×10^{-2} g_{CO2}/g_{pol}. In subplot (d) purple represents the 2100 g/mol system, light blue represents the 5200 g/mol system, green represents the 31000 g/mol system.

Henry's Law Constant

Widom's test particle insertion test was performed on the pure polymer systems and the calculated values for the excess chemical potential were used to evaluate the Henry's law constant with eq (S19):

$$H_i = \frac{\rho RT}{M_i} \lim_{x_i \to 0} \left[\exp\left(-\frac{\mu_i^{\mathrm{ex}}}{RT} \right) \right] \tag{S19}$$

In **Figure S19**, computed values of H_i are compared against experimental values reported by Durill et al. and Sato et al.[S24] In addition, results obtained with the Sanchez-Lacombe EoS are shown. They were calculated using the following expression of the model for the infinite dilution solubility coefficient for a two components system. Based on the definition given in eq (S19), this corresponds to the inverse of the Henry's law constants obtained in the simulations.

$$\ln\frac{1}{H_2} = \ln\left(\frac{T_{STP}}{p_{STP}T}\right) + r_1^0 \left\{ \left[1 + \left(\frac{v_1^*}{v_2^*} - 1 \right) \frac{\rho_1^*}{\rho_1^0} \right] \ln\left(1 - \frac{\rho_1^0}{\rho_1^*} \right) + \left(\frac{v_1^*}{v_2^*} - 1 \right) + \frac{\rho_1^0}{\rho_1^*} \frac{T_1^*}{T} \frac{2}{p_1^*} (1 - k_{12}) \sqrt{p_1^* p_2^*} \right\} \tag{S20}$$

Subscript 1 refers to the polymer, 2 to the gas. All symbols pertaining to the Sanchez-Lacombe EoS were defined in Section 1 of this Supporting Information file. T_{STP} = 273 K and p_{STP} = 101325 Pa. The results obtained with the SL equation of state show a weaker M_w dependence compared to the simulation results.

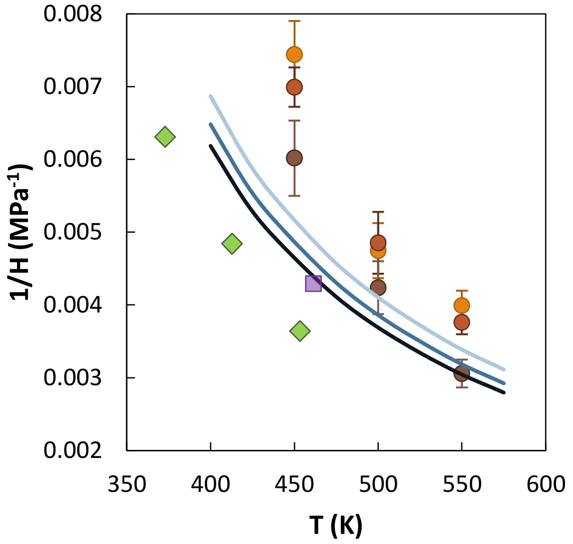

Figure S19. Calculated inverse Henry's law constants for CO_2 solubility in atactic polystyrene in the infinite dilution regime, compared to experimental values of Durrill et al.[S25] (purple square) and Sato et al.[S24] (green diamonds). Circles represent the simulated values: molecular weight of 2100 g/mol is depicted in orange, 5200 g/mol in red and 31000 g/mol in brown. Values calculated with the SL EoS are shown with solid lines: light blue at 2100 g/mol, blue at 3200 g/mol and dark blue at 31200 g/mol.

Enthalpy of Sorption

The values calculated with the Sanchez-Lacombe EoS using k_{ij} with a linear temperature dependence at high temperature deviate from the expected linear trend, whereas, if the k_{ij} values calculated with eq (S16) are used, the trend is linear in the whole temperature range. This finding would suggest that the extrapolation of the values of k_{ij} to higher temperatures using a linear relation might be inaccurate, and therefore it was abandoned.

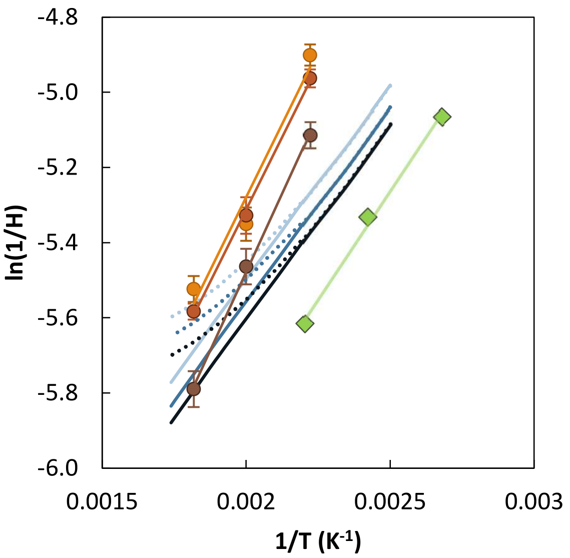

Figure S20. Arrhenius plot for the infinite dilution solubility coefficient for the evaluation of the enthalpy of sorption. Circles represent the simulated values of the inverse of the Henry's law constant defined in eq (S19): molecular weight of 2100 g/mol is depicted in orange, 5200 g/mol in red and 31000 g/mol in brown. Values calculated with the SL EoS are shown with lines: light blue at 2100 g/mol, blue at 3200 g/mol and dark blue at 31200 g/mol. Solid lines are obtained with k_{ij} values calculated using eq (S16), dotted lines with k_{ij} values calculated using a linear extrapolation. Green diamonds are the experimental data of Sato et al.[S24]

Self-diffusion coefficients

Table S10. Self-diffusion coefficients of CO_2 at 450 K, 500 K and 550 K and of aPS at 550 K. In the high M_w case, the polymer did not reach a Fickian diffusion regime in the time of the simulation, therefore the diffusivity could not be extracted.

		**2100 g/mol**	**5200 g/mol**	**31200 g/mol**
$T\ (K)$	$c_{CO_2}(g/g_{pol})$	$D_{aPS,self}$ (m^2/s)	$D_{aPS,self}$ (m^2/s)	$D_{aPS,self}$ (m^2/s)
550	0	4.42×10^{-12}	6.16×10^{-13}	-
	0.005	4.56×10^{-12}	5.45×10^{-13}	-
	0.024	8.33×10^{-12}	1.08×10^{-12}	-
	0.041	1.29×10^{-11}	1.79×10^{-12}	-
$T\ (K)$	$c_{CO_2}(g/g_{pol})$	$D_{CO_2,self}$ (m^2/s)	$D_{CO_2,self}$ (m^2/s)	$D_{CO_2,self}$ (m^2/s)
450	0.007	5.87×10^{-10}	5.28×10^{-10}	3.11×10^{-10}
	0.037	9.11×10^{-10}	7.10×10^{-10}	3.96×10^{-10}
	0.069	1.38×10^{-9}	1.08×10^{-9}	6.46×10^{-10}
500	0.006	1.30×10^{-9}	1.02×10^{-9}	5.86×10^{-10}
	0.028	2.11×10^{-9}	1.68×10^{-9}	8.74×10^{-10}
	0.050	2.71×10^{-9}	2.04×10^{-9}	1.21×10^{-9}
550	0.005	3.30×10^{-9}	2.70×10^{-9}	1.69×10^{-9}
	0.024	4.14×10^{-9}	3.10×10^{-9}	1.99×10^{-9}
	0.041	4.34×10^{-9}	3.88×10^{-9}	2.28×10^{-9}

References

(S1) Müller-Plathe, F. Local Structure and Dynamics in Solvent-Swollen Polymers. *Macromolecules* **1996**, *29* (13), 4782–4791. https://doi.org/10.1021/ma9518767.

(S2) Ndoro, T. V. M.; Voyiatzis, E.; Ghanbari, A.; Theodorou, D. N.; Michael, C. B.; Florian, M. Interface of Grafted and Ungrafted Silica Nanoparticles with a Polystyrene Matrix: Atomistic Molecular Dynamics Simulations. *Macromolecules* **2011**, *44*, 2316–2327. https://doi.org/10.1021/ma102833u.

(S3) Harris, J. G.; Yung, K. H. Carbon Dioxide's Liquid-Vapor Coexistence Curve And Critical Properties as Predicted by a Simple Molecular Model. *J. Phys. Chem.* **1995**, *99* (31), 12021–12024. https://doi.org/10.1021/j100031a034.

(S4) Sanchez, I. C.; Lacombe, R. H. An Elementary Molecular Theory of Classical Fluids. Pure Fluids. *J. Phys. Chem.* **1976**, *80* (21), 2352–2362. https://doi.org/10.1021/j100562a008.

(S5) Lacombe, R. H.; Sanchez, I. C. Statistical Thermodynamics of Fluid Mixtures. *J. Phys. Chem.* **1976**, *80* (23), 2568–2580. https://doi.org/10.1021/j100564a009.

(S6) Sanchez, I. C.; Lacombe, R. H. Statistical Thermodynamics of Polymer Solutions. *Macomolecules* **1978**, *11* (6), 1145–1156.

(S7) Zoller, P.; Walsh, D. *Standard Pressure–Volume–Temperature Data for Polymers*; Technomic- Lancaster, 1995.

(S8) Bevington, P. R.; Robinson, D. K. *Data Reduction and Error Analysis for the Physical Sciences, 3rd Edn.*; McGraw-Hill: Boston, MA, USA, 2003. https://doi.org/10.1063/1.4823194.

(S9) Doghieri, F.; Sarti, G. C. Predicting the Low Pressure Solubility of Gases and Vapors in Glassy Polymers by the NELF Model. *J. Memb. Sci.* **1998**, *147* (1), 73–86. https://doi.org/10.1016/S0376-7388(98)00123-9.

(S10) Sato, Y.; Yurugi, M.; Fujiwara, K.; Takishima, S.; Masuoka, H. Solubilities of Carbon Dioxide and Nitrogen in Polystyrene under High Temperature and Pressure. *Fluid Phase Equilib.* **1996**, *125* (1–2), 129–138. https://doi.org/10.1016/S0378-3812(96)03094-4.

(S11) Doghieri, F.; Sarti, G. C. Nonequilibrium Lattice Fluids: A Predictive Model for the Solubility in Glassy Polymers. *Macromolecules* **1996**, *29* (24), 7885–7896. https://doi.org/10.1021/ma951366c.

(S12) Fox, T. G.; Flory, P. J. The Glass Temperature and Related Properties of Polystyrene. Influence of Molecular Weight. *J. Polym. Sci.* **1954**, *14* (75), 315–319. https://doi.org/10.1002/pol.1954.120147514.

(S13) Quach, A.; Simha, R. Pressure-Volume-Temperature Properties and Transitions of Amorphous Polymers; Polystyrene and Poly(Orthomethylstyrene). *J. Appl. Phys.* **1971**, *42* (12), 4592–4606. https://doi.org/10.1063/1.1659828.

(S14) Ougizawa, T.; Dee, G. T.; Walsh, D. J. PVT Properties and Equations of State of Polystyrene□: Molecular Weight Dependence of the Characteristic Parameters in Equation-of-State Theories. *Polymer (Guildf).* **1988**, *30*, 1675–1679.

(S15) Eslami, H.; Müller-Plathe, F. Molecular Dynamics Simulation of Sorption of Gases in Polystyrene. *Macromolecules* **2007**, *40* (17), 6413–6421. https://doi.org/10.1021/ma070697+.

(S16) Han, J.; Boyd, R. H. Molecular Packing and Small-Penetrant Diffusion in Polystyrene: A Molecular Dynamics Simulation Study. *Polymer (Guildf).* **1996**, *37* (10), 1797–1804. https://doi.org/10.1016/0032-3861(96)87295-8.

(S17) Lyulin, A. V.; Michels, M. A. J. Molecular Dynamics Simulation of Bulk Atactic Polystyrene in the Vicinity of Tg. *Macromolecules* **2002**, *35* (4), 1463–1472. https://doi.org/10.1021/ma011318u.

(S18) Ndoro, T. V. M.; Boehm, M. C.; Müller-Plathe, F. Interface and Interphase Dynamics of Polystyrene Chains near Grafted and Ungrafted Silica Nanoparticles. **2012**, 171–179. https://doi.org/10.1021/ma2020613.

(S19) Fritz, D.; Harmandaris, V. A.; Kremer, K.; Van Der Vegt, N. F. A. Coarse-Grained Polymer Melts Based on Isolated Atomistic Chains: Simulation of Polystyrene of Different Tacticities. *Macromolecules* **2009**, *42* (19), 7579–7588. https://doi.org/10.1021/ma901242h.

(S20) Cotton, J. P.; Farnoux, B.; Jannink, G.; Decker, D.; Benoit, H.; Picot, C.; Higgins, J.; Ober, R.; des Cloizeaux, J. Conformation of Polymer Chain in the Bulk. *Macromolecules* **1974**, *7* (6), 863–872. https://doi.org/10.1021/ma60042a033.

(S21) Wecker, S. M.; Davidson, T.; Cohen, J. B. A Structural Study of Glassy Polystyrene. *J. Mater. Sci.* **1972**, *7* (11), 1249–1259. https://doi.org/10.1007/BF00550690.

(S22) Schubach, H. R.; Nagy, E.; Heise, B. Short Range Order of Amorphous Polymers Derived by WAXS. *COLLOID Polym. Sci. KOLLOID-ZEITSCHRIFT* **1981**, *5* (6), 553–554. https://doi.org/10.1016/j.jemermed.2014.06.025.

(S23) He, Y. Y.; Lutz, T. R.; Ediger, M. D.; Ayyagari, C.; Bedrov, D.; Smith, G. D. NMR Experiments and Molecular Dynamics Simulations of the Segmental Dynamics of Polystyrene. *Macromolecules* **2004**, *37* (13), 5032–5039. https://doi.org/10.1021/ma049843r.

(S24) Sato, Y.; Takikawa, T.; Takishima, S.; Masuoka, H. Solubilities and Diffusion Coefficients of Carbon Dioxide in Poly(Vinyl Acetate) and Polystyrene. *J. Supercrit. Fluids* **2001**, *19* (2), 187–198. https://doi.org/10.1016/S0896-8446(00)00092-9.

(S25) Durrill, P. L.; Griskey, R. G. Diffusion and Solution of Gases in Thermally Softened or Molten Polymers: Part I. Development of Technique and Determination of Data. *AIChE J.* **1966**, *12* (6), 1147–1151. https://doi.org/10.1002/aic.690120619.
